# Malat1 regulates PMN-MDSC expansion and immunosuppression through p-STAT3 ubiquitination in sepsis

**DOI:** 10.7150/ijbs.92267

**Published:** 2024-02-11

**Authors:** Yaodong Wang, Caiyan Zhang, Tingyan Liu, Zhenhao Yu, Kexin Wang, Jiayun Ying, Yao Wang, Ting Zhu, Jingjing Li, Xiuchuan Lucas Hu, Yufeng Zhou, Guoping Lu

**Affiliations:** 1 Department of Critical Care Medicine, Children's Hospital of Fudan University, Shanghai, China.; 2 Shanghai Institute of Infectious Disease and Biosecurity, Fudan University, Shanghai, China.; 3 Institute of Pediatrics, Children's Hospital of Fudan University, National Children's Medical Center, and the Shanghai Key Laboratory of Medical Epigenetics, International Co-laboratory of Medical Epigenetics and Metabolism, Ministry of Science and Technology, Institutes of Biomedical Sciences, Fudan University, Shanghai, China.; 4 National Health Commission (NHC) Key Laboratory of Neonatal Diseases, Fudan University, Shanghai, China.; 5 Fujian Key Laboratory of Neonatal Diseases, Fujian, China.; 6 Lungs for Living Research Centre, UCL Respiratory, University College London, London, UK.

**Keywords:** Experimental sepsis, Polymorphonuclear myeloid-derived suppressor cell, Malat1, STAT3 pathway, Ubiquitination

## Abstract

Myeloid-derived suppressor cells (MDSCs) expand during sepsis and contribute to the development of persistent inflammation-immunosuppression-catabolism syndrome. However, the underlying mechanism remains unclear. Exploring the mechanisms of MDSCs generation may provide therapeutic targets for improving immune status in sepsis. Here, a sepsis mouse model is established by cecal ligation and perforation. Bone marrow cells at different sepsis time points are harvested to detect the proportion of MDSCs and search for differentially expressed genes by RNA-sequence. In lethal models of sepsis, polymorphonuclear-MDSCs (PMN-MDSCs) decrease in early but increase and become activated in late sepsis, which is contrary to the expression of metastasis-associated lung adenocarcinoma transcript 1 (Malat1). *In vivo*, Malat1 inhibitor significantly increases the mortality in mice with late sepsis. And *in vitro*, Malat1 down-regulation increases the proportion of PMN-MDSCs and enhanced its immunosuppressive ability. Mechanistically, Malat1 limits the differentiation of PMN-MDSCs by accelerating the degradation of phosphorylated STAT3. Furthermore, Stattic, an inhibitor of STAT3 phosphorylation, improves the survival of septic mice by inhibiting PMN-MDSCs. Overall, the study identifies a novel insight into the mechanism of sepsis-induced MDSCs and provides more evidence for targeting MDSCs in the treatment of sepsis.

## Introduction

Sepsis is defined as a life-threatening organ dysfunction caused by a dysregulated host response to infection 1. Sepsis affects a fifth of patients admitted to intensive care units (ICUs) with a mortality of 35.5%, indicating a large healthcare and socioeconomic burden 2. In sepsis cases, the host immune response displays an initial excessive inflammatory response to infection that is associated with tissue damage and leads to organ failure and endothelial dysfunction 3. Nowadays, fewer septic patients succumb to early death, due to the advances in modern ICU life support technology. However, a substantial proportion of sepsis survivors will develop persistent inflammation, immunosuppression, and catabolism syndrome (PICS) which may be the result of an inappropriate bone marrow (BM) response. These individuals are predisposed to poor quality of life and indolent death 4,5. The cytokine milieu present during infection drives myelopoiesis at the expense of both erythropoiesis and lymphopoiesis 6-8. Consequently, the BM is composed of nearly 95% myeloid cells within several days of sepsis, and many bone marrow cells (BMCs) that are released in large numbers as immature myeloid cells (IMC) never reach a fully differentiated state 5,9. One fraction of these cells is the myeloid-derived suppressor cells (MDSCs), a population that has been initially described as tumor microenvironment components 10.

MDSCs are heterogeneous and have suppressive effects on innate and adaptive immunity, especially on T cells 11. Although first discovered in cancer patients, subsequent studies highlighted the role of MDSCs in various pathological processes, such as autoimmunity, infectious diseases, and organ transplantation 12. In mice, MDSCs are mainly divided into CD11b^+^Ly6G^-^Ly6C^hi^ monocytic (M)-MDSC and CD11b^+^Ly6G^+^Ly6C^low^ polymorphonuclear (PMN)-MDSC. In humans, PMN-MDSCs correspond to CD33^+^CD11b^+^CD14^-^CD15^+^, while M-MDSCs correspond to CD11b^+^CD14^+^HLA-DR^-/lo^CD15^-^ 13. M-MDSCs and PMN-MDSCs share key biochemical features that enable their suppression of immune responses, for example, the upregulation of signal transducer and activator of transcription 3 (STAT3) expression. They also have unique features that may affect their ability to regulate different aspects of immune responses 14. A 'two-signal' hypothesis has been proposed to describe the expansion and activation of MDSCs 15,16. Additionally, altered epigenetics statuses in sepsis were involved in this process. For example, long non-coding RNA (lncRNA) Hotairm1 supports MDSC repressor function during sepsis in mice 17. The expansion and activation of MDSCs in sepsis involve a variety of regulations, whose underlying mechanisms are not fully understood.

Unlike the well-defined role of MDSCs in cancer, the role of MDSCs in sepsis remains controversial and appears to be time-dependent 18. During sepsis, MDSCs are thought to be beneficial when acutely recruited to inflamed tissues, as they can suppress acute inflammatory responses 18,19. In one study, the adoptive transfer of day 10 MDSCs, rather than day 3, from septic mice into other septic mice had a protective effect 20. However, septic patients with persistent MDSC expansion have a longer ICU stay, greater in-hospital mortality, and are more likely to be discharged to a rehabilitation facility compared with patients for whom MDSC populations return to baseline within 2 weeks 21. Therefore, it appears that sepsis-induced MDSC persistence, rather than initial MDSC expansion, may contribute to PICS pathophysiology 5. In pediatric sepsis, for the first time, Bline et al. 22 described increased MDSCs in children with septic shock, along with an association between the early increase in MDSCs and adverse organ dysfunction outcomes. Previous studies have reported that eliminating MDSCs, targeting MDSC-associated signaling pathways, or preventing MDSC migration may improve the survival of septic mice 23-25. Overall, MDSCs have a dual role in sepsis and are considered important mediators in sepsis immunology. They may serve as promising therapeutic targets for sepsis. However, a more systematic and comprehensive understanding of the molecular mechanisms that govern MDSC development is required.

In this study, to further explore MDSC development, we profiled the expression of mRNA and non-coding RNA in BMCs and identified differential metastasis-associated lung adenocarcinoma transcript 1 (Malat1) expression during the early and late stages of sepsis. Malat1 is a lncRNA that affects immune responses during sepsis and is related to the phenotypes of immune cells such as macrophages 26,27. Previous study have shown that Malat1 expression is associated with circulating MDSCs in lung cancer patients 28. Our results show that Malat1 is involved in the expansion and activation of PMN-MDSCs by affecting the expression of p-STAT3 through the ubiquitin-proteasome system. Studying the Malat1-STAT3-PMN-MDSC axis may provide an underlying therapeutic target for post-acute sepsis correction of immune suppression.

## Materials and Methods

### Patient enrollment, blood collection, and sample preparation

The study was registered with *clinicaltrials.gov* (NCT03996720) and conducted by the Department of Critical Care Medicine at Children's Hospital of Fudan University. Fifteen patients (age < 18 years), diagnosed with sepsis using the Sepsis-3 definitions, eligible for inclusion in the study were enrolled within 12 h of sepsis protocol onset between 2022 and 2023. Late/chronic sepsis was defined as an ICU length of stay greater than or equal to 7 days with evidence of persistent organ dysfunction, measured using components of the Sequential Organ Failure Assessment (SOFA) score. Blood was drawn on day 7 after the sepsis diagnosis. Moreover, control samples were collected from 15 age-matched patients after indirect hernia surgery. Samples were stored at 4 °C and processed within 6 h after the blood draw. Peripheral blood mononuclear cells (PBMCs) were collected using Ficoll-Paque PLUS (GE Healthcare, Chicago, IL, USA) and density gradient centrifugation, and used for flow cytometry analysis or real-time quantitative PCR. All experiments were approved by the Research Ethics Board of the Children's Hospital of Fudan University (approval reference number: EK2022303). Basic patient information is listed in Supplementary Table 1.

### Animals

Male C57BL/6J mice (6 to 8 weeks old) were purchased from Shanghai Jiesijie experimental animal Co., Ltd. All mice were housed in a pathogen-free facility and acclimated to a new environment for 1 week before surgery. All experiments were approved by the Institutional Animal Care and Use Committee of the Children's Hospital of Fudan University (approval reference number: EK2020538).

### Polymicrobial sepsis model

Sepsis was induced by the cecal ligation and puncture (CLP) method in mice as described previously 29. Briefly, a 0.5-cm midline abdominal incision was made, and the cecum was ligated at 2/3 of the upper cecum and punctured twice at the distal side with a 22-gauge needle. A small amount of feces was extruded into the abdominal cavity. The abdominal wall and skin were sutured in layers with 6-0 and 4-0 silk, respectively. Sham-operated mice were treated identically, except that the cecum was neither ligated nor punctured. All mice were subcutaneously injected with 1 ml 0.9% sodium chloride solution for postoperative fluid resuscitation. To establish intra-abdominal infection and approximate the clinical conditions of human sepsis where there is a delay between the onset of sepsis and the delivery of therapy, mice were intraperitoneally injected with antibiotic (Imipenem; 25 mg·kg^-1^ body weight) at 8 and 16 h after surgery. The mortality of CLP group mice within 6 days after the exclusion of manipulative reasons was approximately 60%. All animal experiments were repeated three times, and representative data from one of three mice experiments were presented.

### *In vitro* MDSC induction

The mice were killed by CO_2_ euthanasia. The femurs of mice were taken under aseptic conditions. The BMCs were flushed out of the femurs with PBS (Hyclone Laboratories, Logon, UT, USA). A single-cell suspension was made by pipetting up and down and filtering through a 70-μm nylon strainer, followed by incubation with erythrocyte lysis buffer and washing. Cells were centrifuged at 300 ×g for 5 min at 4 °C. Collected cells were resuspended in RPMI-1640 medium supplemented with 10% fetal bovine serum (FBS) and 1% penicillin and streptomycin (all from Gibco, Grand Island, NY, USA). Cells were cultured in a 24-well plate (1×10^6^ cells per well) and stimulated with granulocyte macrophage-colony stimulating factor (GM-CSF) (20 ng·mL^-1^) and Interleukin (IL)-6 (20 ng·mL^-1^) (both from Sigma-Aldrich, Saint Louis, MO, USA) for 3 days.

### Cell isolation

CD11b^+^Ly6G^+^ cells were isolated from BMCs or the spleen using magnetic beads according to the manufacturer's protocol (Miltenyi Biotech, Auburn, CA, USA). Briefly, the harvested BMCs or MDSCs cultured *in vitro* were obtained as previously described 30. After blocking by FcR blocking reagent, the cell suspension was subjected to a positive selection of the Ly6G^+^ cells by incubating with anti-Ly6G-biotin antibodies for 15 min at 4 °C. Cells were then incubated with anti-biotin magnetic beads for 15 min at 4 °C and subsequently passed through an LS column (Miltenyi). The cells were more than 97% CD11b^+^Ly6G^+^, as determined by flow cytometry.

### Flow cytometry

Isolated cells were resuspended in staining buffer (BD Biosciences, San Jose, CA, USA). After blocking by Fc block Ms CD16/CD32 (Biolegend, San Diego, CA, USA) for 15 min, cells were stained by incubation for 20 min on ice in staining buffer with the following antibodies: anti-CD11b conjugated to FITC (BD), anti-Ly6G conjugated to PE (BD), anti-Ly6C conjugated to PE-Cy7 (Biolegend) for MDSCs, anti-MHC-Ⅱ conjugated to FITC (Biolegend) for MHC-Ⅱ^+^ cells, anti-CD8a conjugated to PE (Biolegend), anti-CD4 conjugated to PE-Cy7 (Biolegend) for T cells. In some experiments, an appropriate isotype-matched control was used for each antibody. After washing, the samples were analyzed by a FACSDiva^TM^ flow cytometer (BD) or a CYTEK^TM^ NL-1000 flow cytometer (Cytek Biosciences, Silicon Valley, CA, USA). About 10,000 events were acquired and analyzed using FlowJo v10 software.

### Real-time quantitative PCR

Real-time quantitative PCR (RT-qPCR) was used to determine the levels of targeted lncRNA and mRNA in cells. Total cellular RNA was isolated from BMCs, CD11b^+^Ly6G^+^ cells, or WBC using TRIzol reagent (Thermo Fisher Scientific, Carlsbad, CA, USA). Then, cDNA was synthesized from 1000 ng of RNA with PrimeScript^TM^ RT reagent Kit with gDNA Eraser (Takara, Dalian, China), according to the manufacturer's instructions. Levels of target RNA expression were measured using QuantiTect PCR. Primers are listed in the Supplementary Table 2. Relative expression levels were calculated using the 2^-ΔΔCT^ cycle threshold method.

### Protein extracts and western blot

For whole cell lysates, the cells were lysed in 1×RIPA buffer (Beyotime Biotechnology, Shanghai, China) containing protease and phosphatase inhibitors (Thermo Fisher Scientific, Carlsbad, CA, USA). After ultrasonic cracking on ice, cell lysates were cleared by centrifugation for 10 min at 4 °C and 12, 000 ×g. Protein concentrations were determined by the BCA method, and protein samples were kept at -80 °C. Protein extracts or immunoprecipitated protein complexes were subjected to electrophoresis using 10% SDS-polyacrylamide gel (Beyotime Biotechnology, Shanghai, China) and transferred to nitrocellulose membranes (Sigma-Aldrich, Saint Louis, MO, USA). Membranes were blocked with a protein-free rapid blocking solution (Epizyme Biomedical Technology, Shanghai, China) for 20 min at room temperature, and then probed overnight at 4 °C with an anti-targeted protein antibody (Supplementary Table 3). After washing with TBST (Epizyme), blots were incubated with appropriate HRP-conjugated secondary antibodies for 1 h at room temperature. Proteins were detected with the enhanced chemiluminescence detection system (Thermo Fisher Scientific, Carlsbad, CA, USA), the bands were visualized using LAS-4000IR, and the gray value of the bands was analyzed with Image J software.

### RNA sequencing

BMCs were isolated from controls, CLP day 1 mice, and CLP day 6 mice. The RNA of the total sample was isolated and purified by Trizol, and RNA integrity was detected using a Bioanalyzer 2100 (Agilent Technologies). Oligo (dT) magnetic beads were used to specifically capture the mRNA containing PolyA through two rounds of purification, and then the mRNA was fragmented and reversely transcribed. E. coli DNA polymerase I and RNase H were synthesized by adding dUTP Solution into the two strands to complete the ends of the double-stranded DNA into flat ends. Then, an A base is added to each of its ends, and magnetic beads are used to screen and purify the fragment size. The two strands were digested with UDG enzyme, then PCR-pre denatured at 95 °C for 3 min, denatured at 98 °C for a total of eight cycles of 15 s each, annealed at 60 °C for 15 s, extended at 72 °C for 30 s, and finally maintained at 72 °C for 5 min to form a library of about 300 bp. Finally, they were sequenced using Illumina NovaSeq 6000 in PE 150 mode, and the results were analyzed by DESseq2 software (accomplished by LC Sciences, Hangzhou, China). RNA sequencing data were deposited in the GEO database under the accession number GSE222808.

### T-cell suppression assay

To determine the suppressive effects of CD11b^+^Ly6G^+^ cells on T cells, we co-cultured them and tested the proliferation of T cells. Firstly, 1 mL PBS containing 2 μg·mL^-1^ anti-CD3 antibody was placed in a 24-well plate for incubation for 4 h at 37 °C. Splenocytes were isolated from healthy control mice and filtered through a 70-μm nylon strainer to obtain a single-cell suspension. Then, cells were labeled with carboxy-fluorescein diacetate, succinimidyl eater (CFSE) through incubation for 15 min in a 37 °C water bath with 5 μM CFSE dye (BD Biosciences, San Jose, CA, USA). The labeled splenocytes were co-cultured in a 1:1 or 1:2 ratio with isolated CD11b^+^Ly6G^+^ cells in the 24-well plate coated with anti-CD3 antibody. Anti-CD28 antibody (2 μg·mL^-1^) were added to the culture to stimulate T cell proliferation. After 3 days, the cells were harvested and CD4^+^ or CD8^+^ T cell proliferation was determined by the stepwise dilution of CFSE dye in dividing T cells using flow cytometry.

### Malat1 expression plasmid

Malat1 was cloned in GV712 expression vector (CMV enhancer-MCS-SV40-puromycin) downstream of the CMV promoter by Genechem (Shanghai, China). An empty control vector served as a negative control.

### Cell transfection

BMCs or PMN-MDSCs were transfected with electroporation according to the manufacturer's protocol (Celetrix Biotechnologies, Manassas, VA, USA). Briefly, for Malat1 knockdown, Malat1-specific or scrambled lncRNA smart silencer (Ribo, Guangzhou, China) (2 μM each) was suspended in 125 μL reagent containing 5×10^6^ cells. For Malat1 overexpression, Malat1 plasmid (1.5 μg) was suspended in 125 μL reagent. The condition of electroporation is 750 V and 20 ms. After transfecting, cells were cultured in RPMI-1640 medium GM-CSF for 48 h. To maintain the cells survival, GM-CSF (5 ng·mL^-1^) was added into the culture. Sequences of Malat1-specific or scrambled lncRNA smart silencer (Ribo, Guangzhou, China) were listed in the supplementary Table 2.

### Immunoprecipitation

The cells in each group (approximately 5×10^6^) were lysed in 1×RIPA buffer containing protease and phosphatase inhibitors (Thermo Fisher Scientific, Carlsbad, CA, USA) to obtain protein extracts. p-STAT3 antibody (CST, 1:100) was added to protein extracts, which were incubated using a rotary mixer at 4 °C overnight. Then, the protein-antibody complex was incubated with protein A/G magnetic beads (50 μL) using a rotary mixer at 4 °C for 3 h. Magnetic bead-bound complexes were immobilized with magnets. Ubiquitin in p-STAT3 proteins was detected by western blot.

### Ubiquitination assay

After 48 h of transfection, 20 μM of MG-132 (Selleck Chemicals, Houston, TX, USA) or an equal concentration of DMSO was added to the medium for 4 h, followed by protein extraction for western blot analysis. Cell lysates were immunoprecipitated with the labeled antibodies and overnight incubated at 4 °C. The eluted proteins were determined by western blot.

### Luminex assay

Cytokines and chemokines from plasma of sham or CLP mice, including IL-4, IL-6, IL-10, IL-17, tumor necrosis factor alpha (TNF-α), and C-C motif chemokine ligand 2 (CCL2), were quantified by Luminex (Performed at Universal Biotech, Shanghai, China).

### ELISA

Enzyme-linked immunosortbent assay (ELISA) kits were used to measure the levels of IL-6 and IL-10 (ABclonal, Boston, MA, USA) in the serum of mice according the manufactory manual. Samples were run in duplicate.

### *In vitro* and *in vivo* treatment

PMN-MDSCs were isolated as described in the cell isolation section, and mice received 1×10^6^ PMN-MDSCs by intravenous (*i.v.*) injection immediately after CLP. Control mice were treated with equal volumes of PBS. We used Malat1-IN-1 (MedChemexpress, New Jersey, NJ, USA) to regulate the downstream genes of Malat1. And we used Stattic (Selleck Chemicals, Houston, TX, USA) to inhibit the phosphorylation of STAT3 on Tyr705. In cell differentiation experiments, 5 μM Stattic or an equal concentration of Vehicle (DMSO) was added to the medium for 48 h. In the studies of cell function, 5 μM Stattic or an equal concentration of Vehicle was added to the medium for 6 h, followed by the T cell suppression assay. In animal experiments, 5 mg·kg^-1^ Stattic or an equal concentration of Vehicle was intraperitoneally injected once before CLP and once two days after CLP.

### Bacterial count

Whole blood and crushed spleen were plated in serial log dilutions on tryptone soya agar plates (Beijing Land Bridge Technology, Beijing, China). After plating, tryptone soya agar plates were incubated at 37 °C aerobically for 24 h. Results are expressed as CFU per milliliter of blood or per gram of spleen.

### Statistical analysis

For comparisons among multiple groups, one-way ANOVA was applied. The independent sample t-test or the Mann-Whitney U test was used for the comparisons between the two groups. Results are shown as mean ± standard error of the mean (SEM). All analyses were performed using GraphPad Prism 8 software. Differences were considered significant at a *p*-value of < 0.05.

## Results

### The immune status of CLP-induced septic mice changed from a hyperinflammatory response to immunosuppression

We established the sepsis model using the CLP method, which is considered to be the gold standard model in sepsis research because it imitates the pathological development of sepsis 31. The cecum was ligated and punctured in CLP surgery on day 0 (Fig. S1A). CLP mice developed diarrhea, giant spleens (Fig. S1B), and other symptoms within a few days after resuscitation and had a higher mortality rate than sham mice. Organ injuries were assessed by HE staining, tissue damage to the lung and liver was gradually aggravated, and immune cell infiltration increased with the progression of CLP (Fig. S1C). HE staining showed that at least two important organs, the lung, and liver, were injured after CLP, which conformed to the definition of sepsis 3.0, according to the Third International Consensus Definitions for Sepsis and Septic Shock 1.

To evaluate the immune status of sepsis in the CLP model, plasma cytokines and chemokines were measured by the Luminex microbead-based multiplex assay at different stages of sepsis. As expected, IL-4, IL-6, IL-10, IL-17, TNF-α, and CCL2 were significantly elevated from the early stage of sepsis, but on CLP day 6, all cytokines were reduced to levels similar to those of sham mice. On CLP day 10, IL-4 and IL-17 increased again, and the levels of IL-6 were consistently higher than those in sham mice at the time points we examined, indicating a persistent low-grade inflammatory response (Fig. S1D). Considering that mRNA may reflect the state of immune cells in a more timely manner, we found that the level of IL-6 mRNA in circulating WBC was significantly decreased while that of IL-10 mRNA still increased on CLP day 6 (Fig. S1E). The mRNA levels of IL-6 in BMCs were also lower than those in sham mice on CLP day 6 (Fig. S1F). CLP-induced sepsis imitate the transition from excessive inflammation to immunosuppression. Recent studies revealed that the period after day 5 of CLP has been considered as the immunosuppressive period, in which mice are more likely to suffer secondary infections 32-34. Combining previous studies and our own findings, we tend to defined CLP day 6 as late sepsis in this study.

### MDSCs increase in the late stage of sepsis and mediate immune suppression

**Figure 1:**
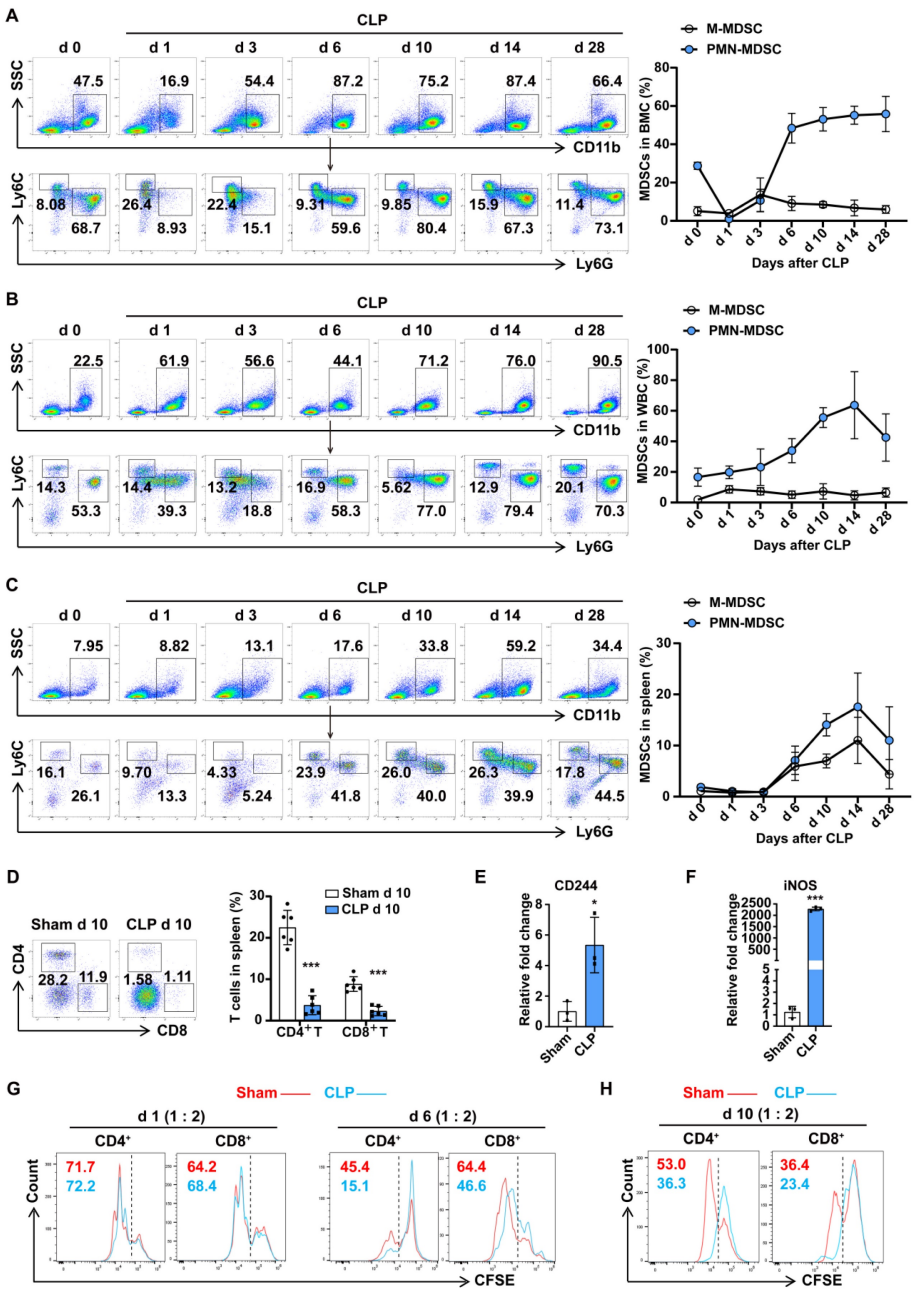
** PMN-MDSC expand and persist in the late stage of sepsis.** (A-C) PMN-MDSCs and M-MDSCs in BMCs, WBCs, and spleen were quantified by flow cytometry. Representative flow cytometry plots and the statistical graph show the percentages on days 0, 1, 3, 6, 10, 14, 28 post-CLP. n=6. (D) Splenocytes were isolated from sham and CLP day 10 mice, and MDSCs, CD4^+^ T cells, and CD8^+^ T cells were detected by flow cytometry. Representative dot plots and the statistical graph show the percentage of MDSC subsets and T cells in the spleen on day 10 post-CLP. n=6. (E-F) RT-qPCR analysis of CD244 and iNOS expression in CD11b^+^Ly6G^+^ cells derived from BMCs of sham and CLP mice on day 6. n=3, each sample was a mixture of 3 mice. (G) Splenocytes were isolated from healthy control mice and labeled with the fluorescent dye CFSE. CD11b^+^Ly6G^+^ cells isolated from BMCs of sham and CLP mice on days 1 and 6 were co-cultured with splenocytes in a ratio of 1:2 for 3 days. T cell proliferation was determined by the stepwise dilution of CFSE dye in dividing T cells using flow cytometry. Representative flow cytometry plots of CFSE-positive and low-positive T cells gated on CD4 and CD8 are shown. (H) CD11b^+^Ly6G^+^ cells were isolated from the spleen of sham and CLP mice on day 10 and co-cultured with CFSE-labeled splenocytes (1:4 ratio) for 3 days. T cell proliferation was detected by flow cytometry. Data are presented as means ± SEM. *p < 0.05, **p < 0.01, ***p < 0.001.

MDSCs may be involved in immunosuppression during sepsis, and the number of MDSCs was an important parameter. Compared with trauma controls, the percentages of PMN-MDSCs in the PBMCs of septic patients were significantly higher, by up to over 60%, while no significant change in M-MDSCs was observed (Fig. S2A). To understand whether MDSCs contribute to the development of polymicrobial sepsis, we administered CLP to mice and examined MDSCs in BMCs, and circulating leukocytes in surviving mice by flow cytometry. As shown in Fig. 1A, PMN-MDSCs initially showed a transient decrease in the bone marrow on day 1 post-CLP, followed by a gradual increase until reaching a peak level (55%) by day 6, which persisted until day 28; while in the peripheral blood, PMN-MDSCs did not show significant changes before or immediately after CLP, but began to markedly rise three days later and peaked at 60% on day 14, with some recovery observed by day 28, while MHC-Ⅱ^+^ cells showed the opposite trend (Fig. 1B, S2B); M-MDSCs exhibited no significant dynamics regardless of location. In addition to secreting proteins that mediated immunosuppression, MDSCs can also migrate to other tissues or organs and suppressive immune cells by direct contact. The spleen, as the most important organ for antibacterial and antifungal immune reactivity, plays an important role in the immune regulating process during sepsis. To investigate the infiltration of MDSCs in spleen dysfunction during sepsis, we examined the accumulation of splenetic MDSCs and lymphocytes. The dynamic changes of PMN-MDSCs in the spleen were consistent with PMN-MDSCs in the peripheral blood, with an initial increase starting three days post-CLP, reaching a peak level (15%) on day 14, and then declining below the pre-day 10 levels by day 28, whereas the percentages of both CD4^+^ and CD8^+^ T cells significantly decreased (Fig. 1C, D). These findings suggest that the populations of PMN-MDSCs and M-MDSCs undergo distinct temporal patterns following CLP, with PMN-MDSCs showing a more prolonged response than M-MDSCs.

To better understand whether PMN-MDSCs could contribute to sepsis-induced immune dysfunction, we isolated PMN-MDSCs (Ly6G^+^CD11b^+^) from BMCs or splenocytes (sorting efficiency >97%, Fig. S2C). The expression of CD244 has been confirmed to be useful for identifying PMN-MDSCs and neutrophils. PMN-MDSCs isolated from the BM on late sepsis had higher mRNA levels of CD244 and inducible nitric oxide synthase (iNOS) (Fig. 1E, F). The activation of STAT3 and elevated protein level of iNOS were also detected in PMN-MDSCs from mice with late sepsis (Fig. S2D). MDSCs are reported to contribute to the suppression of different cells of the immune system. However, the inhibition of T cells is the 'gold' standard for the evaluation of MDSC function, and the inhibition of T-cell activity appears to be sufficient for the designation of cells as MDSCs. To achieve this, we co-cultured PMN-MDSCs isolated from the BM in early and late sepsis with splenocytes. As shown in Fig. 1G and Fig. S2E, PMN-MDSCs from late sepsis significantly inhibited the proliferation of CD4^+^ and CD8^+^ T cells, whereas CD11b^+^Ly6G^+^ cells from early sepsis could not. Consistently, we observed that PMN-MDSCs from the spleen of CLP day 10 mice also inhibited T cell proliferation (Fig. 1H). Collectively, these results demonstrated that PMN-MDSCs expanded and mediated immunosuppression in the late stage of sepsis.

### Malat1 expression is decreased in late sepsis

**Figure 2:**
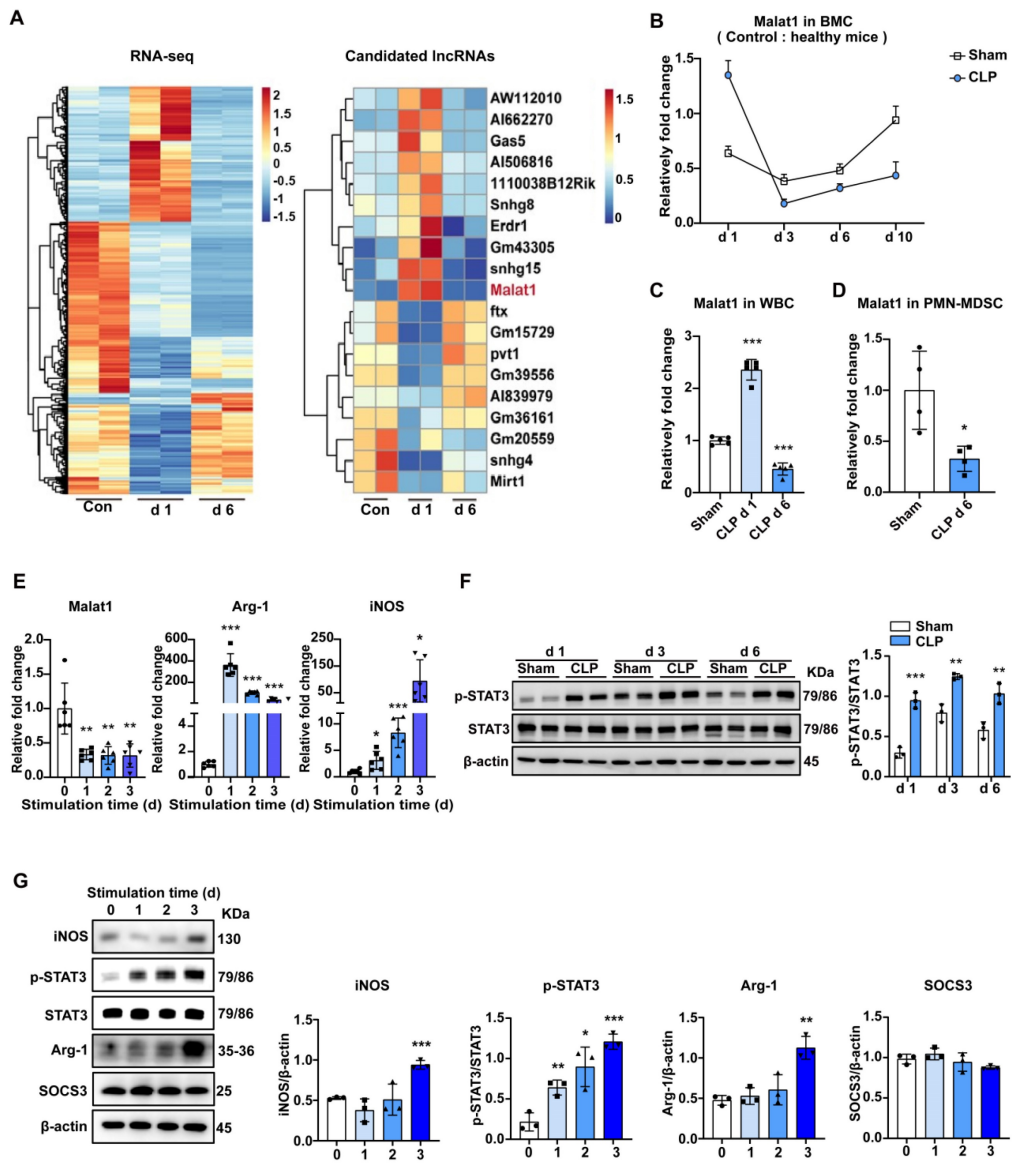
** Malat1 is downregulated in the late stage of sepsis with enhanced STAT3 phosphorylation.** (A) BMCs were isolated from healthy controls, CLP day 1 mice, and CLP day 6 mice. The clustered heatmap showed the top 19 differentially expressed lncRNAs. n = 2, each sample was a mixture of 3-4 mice. (B) RT-qPCR analysis of Malat1 expression in BMCs of sham and CLP mice. n=3-7. (C) RT-qPCR analysis of Malat1 expression in WBCs of sham and CLP mice. n = 5, each sample was a mixture of 2 mice. (D) RT-qPCR analysis of Malat1 expression in PMN-MDSCs of sham and CLP mice on day 6. n = 4, each sample was a mixture of 3 mice. (E) Fresh BMCs were isolated and cultured with GM-CSF plus IL-6 *in vitro*. RT-qPCR analysis of Malat1, Arg-1, and iNOS expression in induced BMCs. (F) STAT3 protein phosphorylation assessment on days 1, 3, and 6 in BMCs of sham and CLP mice (Top) and quantification analysis (Bottom). n=3. (G) p-STAT3, SOCS3, Arg-1, and iNOS protein expression in BMCs after GM-CSF and IL-6 induction (Top) and quantification analysis (Bottom). Data are presented as means ± SEM. *p < 0.05, **p < 0.01, ***p < 0.001.

There are no clear boundaries between the different stages of sepsis development. The specific mechanism of the regular differentiation of PMN-MDSCs in this process remains unclear, but it may be accompanied by the coordination and regulation of epigenetic markers and gene transcriptional activity. To fully reveal the development of PMN-MDSCs at different stages of sepsis, RNA-sequencing (RNA-seq) was performed on BMCs of healthy controls, CLP day 1 mice, and CLP day 6 mice. There were significant differences in gene expression profiles between the three groups, which could be used to distinguish early and late sepsis. The gene expression of BMCs was dramatically changed in early sepsis, and although the gene expression profile in the late stage of sepsis was somewhat similar to that of healthy controls, many differences existed (Fig. 2A).

LncRNAs, acting as crucial regulators of cell biology, have attracted increasing attention. Study have shown that lncRNAs can regulate cell development and immune responses through lineage-specific expression 35. RNA-seq data showed that LncRNA Malat1 expression was elevated in early sepsis and decreased in late sepsis, compared to that in BMCs under physiological conditions, which was contrary to the change in the ratio of PMN-MDSCs among BMCs. In the subsequent experiments, we first confirmed the RNA-seq results by qRT-PCR (Fig. 2B). Specifically, Malat1 expression increased in BMCs within 24 h of CLP, and achieved its lowest level on day 3. Later, Malat1 levels in CLP mice recovered slowly but were still lower than those in sham groups. A similar trend in Malat1 and elevated CD244 mRNA on day 6 in WBCs of septic mice was observed (Fig. 2C, S3A). Moreover, low Malat1 expression was detected in PMN-MDSCs isolated from the BM in late sepsis (Fig. 2D) and in PBMCs isolated from septic patients (Fig. S3B).

To further confirm the pattern of Malat1 expression in sepsis-induced PMN-MDSCs, we performed the induction of MDSCs from BMCs *in vitro* and investigated the activation and function of MDSCs, as well as the expression of Malat1. Under the stimulation of GM-CSF and IL-6, the proportion of PMN-MDSCs in BMCs increased gradually (Fig. S3C). After induction, mRNA levels of arginase 1 (Arg-1) and iNOS increased, while Malat1 expression decreased (Fig. 2E).

### GM-CSF and IL-6-induced PMN-MDSCs may mimic sepsis-derived PMN-MDSC in vitro

Studies have shown the several strategies about inducing marrow cells into MDSCs^36^. Here, we performed the induction of MDSCs from BMCs by GM-CSF and IL-6 *in vitro*. After stimulation, PMN-MDSCs in BMCs increased gradually with mRNA levels of Arg-1, iNOS increased, and Malat1 expression decreased (Fig. 2E). PMN-MDSCs induced *in vitro* could also significantly inhibit the proliferation of T cells (Fig. S3D).

STAT3 activation promotes MDSC expansion. In BMCs, the expression levels of phosphorylated STAT3 in the sepsis group were higher than those in the sham group on days 1, 3, and 6 (Fig. 2F). Perhaps due to the inflammatory response caused by surgical trauma, STAT3 was also slightly activated in the sham group. STAT3 activation was most significant in both CLP and sham groups on day 3. Similar to sepsis-derived MDSCs, the protein level of p-STAT3 also increased gradually while suppressors of cytokine signaling 3 (SOCS3) which was an inhibitor of STAT3 pathway did not change significantly in induced MDSCs (Fig. 2G, S3E). The protein levels of Arg-1 and iNOS were not increased until the third day after stimulation (Fig. 2G).

Derive et al. 20 have found that MDSCs from CLP mice confer protection during sepsis through the promotion of bacterial killing. Here, adoptive transfer of induced PMN-MDSCs also improved bacterial clearance and significantly alleviated septicemia (Fig. S3F, G). Additionally, PMN-MDSCs treated mice had lower proportions of CD11b^+^Ly6G^+^ cells in circulation and higher proportions in BMCs, which may suggest a weaker mobilization of BM-PMN in early sepsis (Fig. S4A, B). When examining long-term outcomes after PMN-MDSCs treatment for early sepsis, we found PMN-MDSCs in circulation and BMCs were significantly reduced, and CD4^+^ and CD8^+^ T cells were partly elevated (Fig. S4C-E). Finally, HE staining showed that PMN-MDSCs treatment alleviated lung and liver damage in early and late sepsis (Fig. S4F), further confirming its effect. Overall, GM-CSF and IL-6-induced PMN-MDSCs have similar phenotypes and functions to sepsis-derived PMN-MDSCs, which may be suitable for this study.

### Malat1 inhibitor aggravated the severity of mice with late sepsis

**Figure 3:**
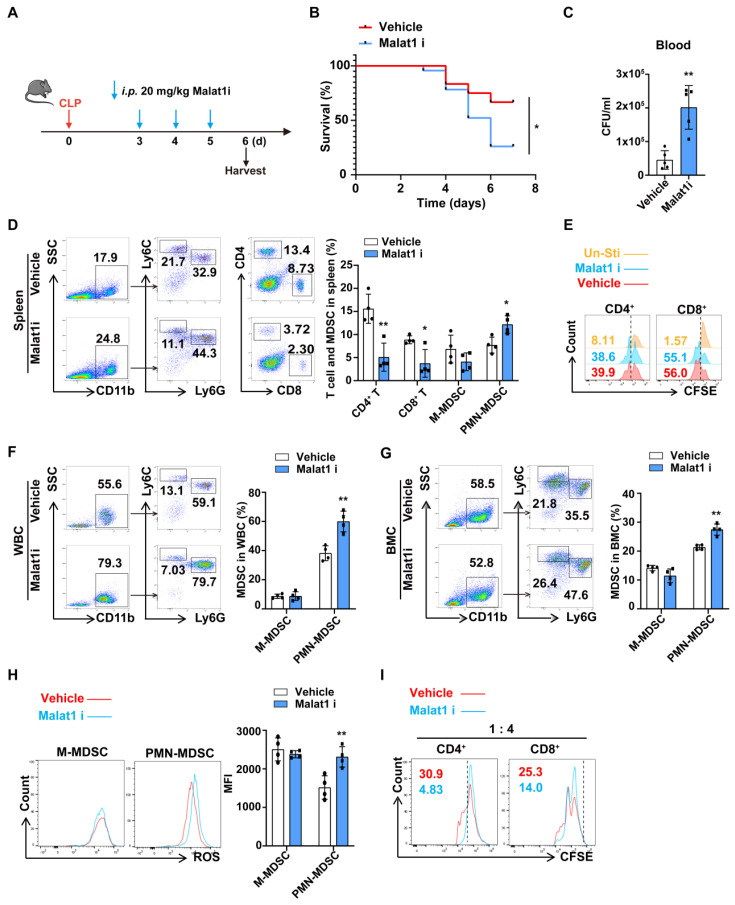
** Malat1 inhibitor aggravated the severity of sepsis by promoting PMN-MDSCs expansion.** (A-F) Injection of Malat1 inhibitor in CLP mice. (A) Schematic illustration of administration protocol. (B) 7-day survival curves of CLP mice treated with Malat1i (n = 24) or Vehicle (n = 12). (C) Results of bacterial counts in whole blood are expressed as CFU per mL of blood. n=5. (D) Representative flow cytometry plots (Left) and the statistical graph (Right) show the percentage of MDSCs and T cells in spleen. n=4. (E) CFSE-labeled splenocytes were treated with vehicle or 5 μM Malalt1 inhibitor. T cell proliferation was detected by flow cytometry. (F) Representative flow cytometry plots (Left) and the statistical graph (Right) show the percentage of MDSCs in circulation. (G-I) Induced MDSC were treated with Malat1 inhibitor for 3 days. (G) Representative flow cytometry plots (Left) and the statistical graph (Right) show the percentage of MDSCs in BMC. (H) Production of ROS in MDSCs was detected by flow cytometry. (I) T cell proliferation was detected by flow cytometry. Data presented as means ± SEM. *p < 0.05, **p < 0.01.

Since both BMCs and PMN-MDSCs have low Malat1 expression during late sepsis, we questioned whether there is a causal relationship between decreased Malat1 expression and PMN-MDSC expansion. MALAT1-IN-1, a potent and specific Malat1 inhibitor, modulated Malat1 downstream genes in a dose-dependent manner^37^. CLP mice were randomly divided into vehicle and Malat1i (intraperitoneally injected with MALAT1-IN-1) groups (Fig. 3A). Malat1 inhibitor appeared to impair bacteria clearance, resulting in more severe bacteremia and higher mortality in late sepsis (Fig. 3B, C). And mice in Malat1i group tend to have lower proportions of CD4^+^ and CD8^+^ T cells but more CD11b^+^Ly6G^+^ Cells in speen (Fig. 3D). In vitro, Malat1 inhibitor had no effect in T cell proliferation, suggesting that the reduction in T cells was not a result of this inhibitor (Fig. 3E). Additionally, mice in Malat1i group also had higher proportions of CD11b^+^Ly6G^+^ Cells in circulation (Fig. 3F). In* vitro*, Malat1 inhibitor increased the proportion of BM-induced PMN-MDSCs and their production of reactive oxygen species (ROS) but had no significant effects on M-MDSC (Fig. 3G, H). PMN-MDSCs treated with Malat1 inhibitor also had a more robust inhibitory effect on T cell proliferation (Fig. 3I). These results indicated reduction of Malat1 in late sepsis may aggravate the severity through promoting the expansion of PMN-MDSCs.

### Malat1 suppressed the differentiation and immunosuppression of PMN-MDSCs by disrupting STAT3 signaling

**Figure 4:**
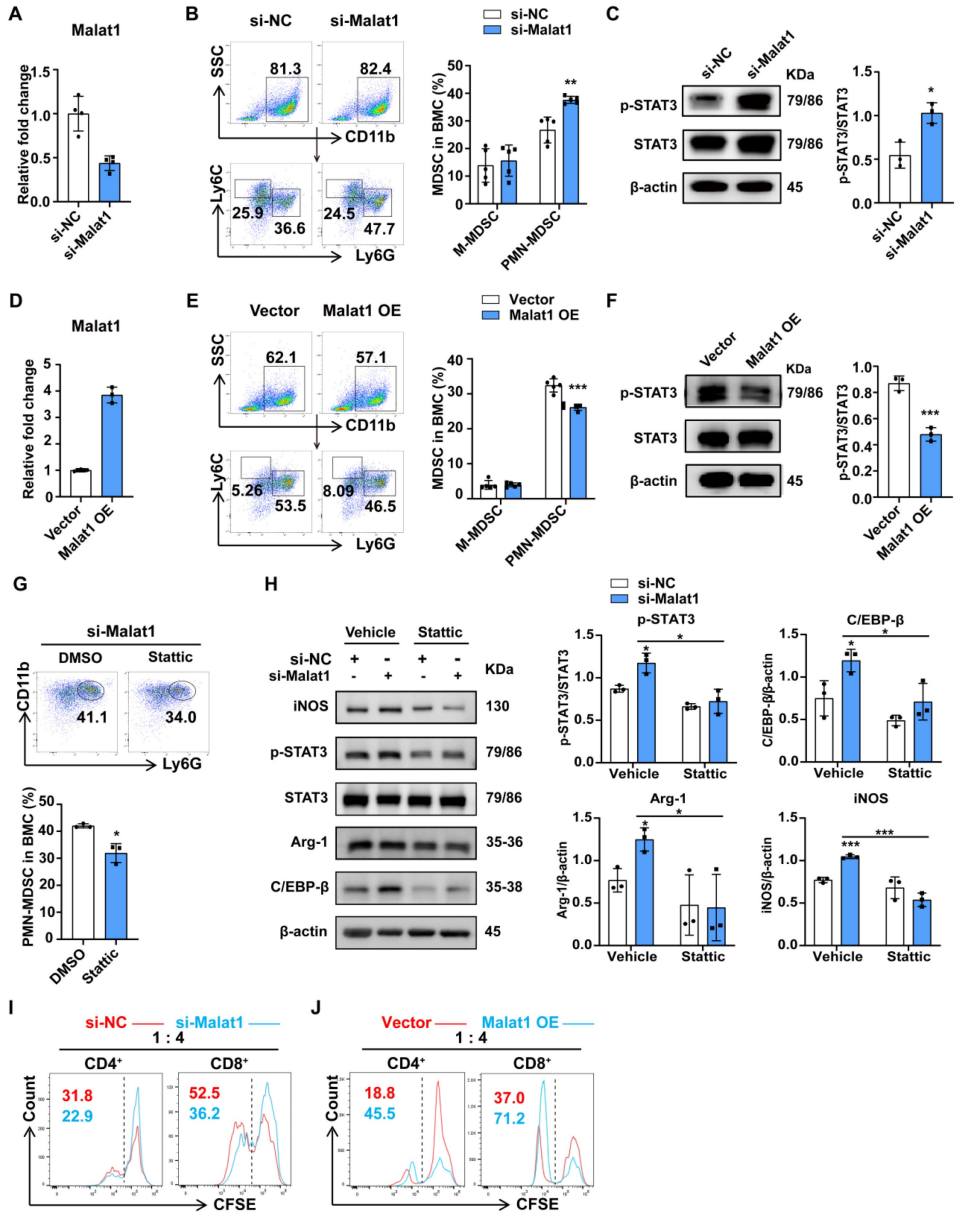
** Malat1 inhibits the expansion and immunosuppressive function of PMN-MDSC by reducing STAT3 phosphorylation.** (A-C) Fresh BMCs were transfected with Malat1-specific or scramble siRNA and ASO for 36 h and then treated with GM-CSF and IL-6 for another 36 h. (A) RT-qPCR analysis of Malat1 expression in BMCs at the first 36 h after transfection. (B) Representative dot plots and the statistical graph show the percentages of MDSC subsets after Malat1 knockdown. (C) STAT3 protein phosphorylation assessment in BMCs after Malat1 knockdown (Left) and quantification analysis (Right). (D-F) Fresh BMCs were transfected with Malat1 expression plasmid or empty control vector for 36 h and then treated with GM-CSF and IL-6 for another 36 h. (D) RT-qPCR analysis of Malat1 expression in BMCs at the first 36 h after transfection. (E) Representative dot plots and the statistical graph show the percentage of MDSC subsets after Malat1 overexpression. (F) STAT3 protein phosphorylation assessment in BMCs after Malat1 overexpression (Left) and quantification analysis (Right). (G) BMCs with Malat1 knockdown were treated with GM-CSF and IL-6 in the presence of Stattic or DMSO for 36 h. Representative dot plots (Top) and the statistical graph (Bottom) show the percentage of PMN-MDSCs. (H) BMCs were treated with 5 μM Stattic or an equal concentration of vehicle for 24 h after Malat1 knockdown, protein expressions were evaluated. (I) PMN-MDSCs with or without Malat1 knockdown were co-cultured with CFSE-labeled splenocytes (1:4 ratio) for 3 days. T cell proliferation was detected by flow cytometry. (J) PMN-MDSCs with or without Malat1 overexpression were co-cultured with CFSE-labeled splenocytes (1:4 ratio) for 3 days. T cell proliferation was detected by flow cytometry. Data are presented as means ± SEM. *p < 0.05, **p < 0.01.

To further determine whether Malat1 could influence the differentiation of PMN-MDSCs, siRNA and antisense oligonucleotides (ASO) were transfected into BMCs, and the proportions of MDSCs among BMCs were then evaluated. The knockdown efficiency was confirmed (Fig. 4A). Flow cytometry revealed that Malat1 knockdown significantly promoted the generation of PMN-MDSCs rather than M-MDSCs (Fig. 4B). Western blot assays showed that Malat1 knockdown enhanced STAT3 activation but did not affect SOCS3 expression (Fig. 4C, S5A). To further confirm these results, BMCs were transfected with Malat1 expression plasmid (GV712) and then induced with GM-CSF and IL-6. Malat1 levels were assessed by qPCR (Fig. 4D). In contrast, Malat1 upregulation decreased the proportion of PMN-MDSCs in BMCs and inhibited the expression of p-STAT3 (Fig. 4E, F). Next, we examined whether p-STAT3 inhibition could rescue the Malat1 knockdown-induced expansion and immunosuppression of PMN-MDSCs. Stattic, an inhibitor of STAT3 phosphorylation on the Tyr 705 site, was incubated with BMCs after the transfection of a Malat1 siRNA and ASO cocktail. Inhibition of STAT3 phosphorylation by Stattic attenuated the promotion effect of Malat1 knockdown on PMN-MDSC expansion, which further indicated that Malat1 affected PMN-MDSC expansion at least partly through the p-STAT3 pathway (Fig. 4G). In addition, we monitored changes in levels of molecules previously reported to be involved in PMN-MDSC expansion, including C/EBP family, Irf4, and Irf8 15,38. Notably, lowering Malat1 expression contributed to increased C/EBPβ expression levels without affecting the others, and stattic treatment restored C/EBPβ levels (Fig. 4H, S5B, C), suggesting that the Malat1-STAT3 signaling axis controlled PMN-MDSC expansion by modulating the activity of the transcription factor C/EBPβ.

Considering that PMN-MDSCs from late septic mice with low Malat1 expression had stronger immunosuppressive ability than those from the sham group, we hypothesized that Malat1 could also affect PMN-MDSC suppressive functions. PMN-MDSCs with Malat1 knockdown were co-cultured with spleen cells from healthy mice. The T-cell suppression assay showed that Malat1 knockdown enhanced the inhibitory effects of PMN-MDSCs on CD4^+^ and CD8^+^ T cell proliferation (Fig. 4I). Conversely, Malat1 overexpression attenuated the immunosuppressive function of PMN-MDSCs (Fig. 4J). Collectively, these data suggest that the sepsis-induced decrease in Malat1 expression promotes the expansion and immunosuppression of PMN-MDSCs by enhancing STAT3 phosphorylation.

### Malat1 inhibits STAT3 phosphorylation by enhancing p-STAT3 protein ubiquitination

The above data showed that sepsis-induced Malat1 downregulation resulted in p-STAT3-dependent expansion and the immunosuppression of PMN-MDSCs. However, the specific mechanism through which Malat1 regulates STAT3 phosphorylation remains unknown. Our data showed that STAT3 was significantly activated from CLP day 1, while Malat1 expression changed with the progression of sepsis, suggesting that Malat1 may not directly affect STAT3 activation. Therefore, we first focused on SOCS3, an inhibitor of STAT3, but no significant changes in SOCS3 expression were observed both in the CLP model (Fig. S3E) and the cell experiments *in vitro* (Fig. 2G and S5A). According to Zhang et al.^39^, lncRNA MEG3 binds to p-STAT3 in cervical cancer cells, resulting in p-STAT3 ubiquitination and degradation. On day 3 of surgery, we found that the activation of STAT3 was the highest when Malat1 expression was the lowest in the CLP group. Thus, we wondered whether Malat1 controls STAT3 signaling by regulating the ubiquitination of p-STAT3.

**Figure 5:**
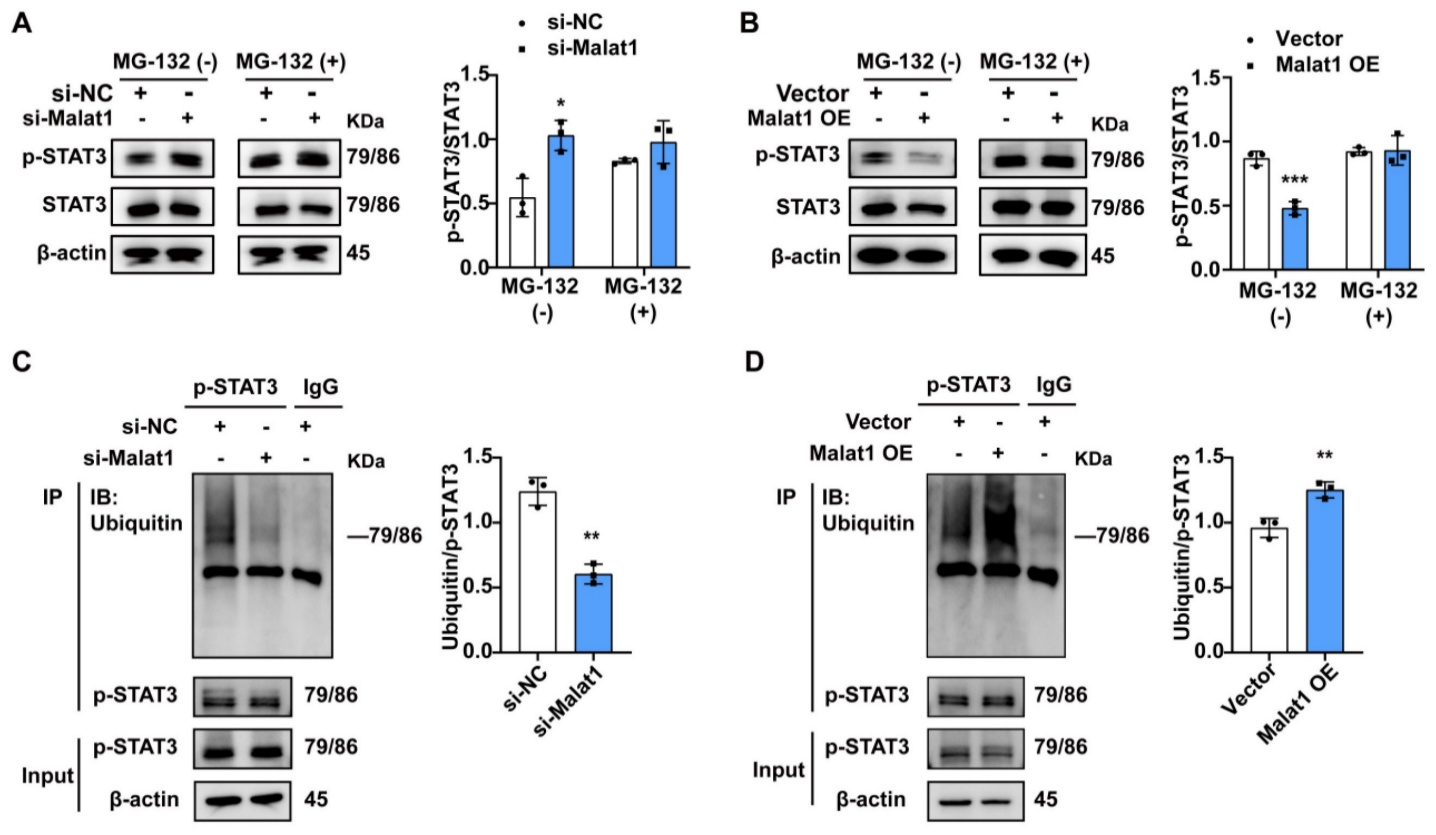
** Malat1 attenuates the level of p-STAT3 protein by promoting its ubiquitination degradation.** (A) Induced PMN-MDSCs with Malat1 knockdown were cultured in the presence or absence of MG132 for 4 h and then subjected to immunoblotting analysis with a p-STAT3 antibody (Left). The statistical graph (Right) shows the quantification analysis. (B) Induced PMN-MDSCs with Malat1 overexpression were cultured in the presence or absence of MG132 for 4 h and then subjected to immunoblotting analysis with a p-STAT3 antibody (Left). The statistical graph (Right) shows the quantification analysis. (C) Ubiquitin level assessment (Left) by western blotting after immunoprecipitation with p-STAT3 or IgG antibody in NC- or Malat1 knockdown PMN-MDSCs. The statistical graph (Right) shows the level of ubiquitination on p-STAT3 protein after Malat1 knockdown. (D) Ubiquitin level assessment by western blotting after immunoprecipitation with p-STAT3 or IgG antibody in empty vector or Malat1 overexpression PMN-MDSCs. The statistical graph (Right) shows the level of p-STAT3 protein ubiquitination after Malat1 overexpression. Data are presented as means ± SEM. *p < 0.05, **p < 0.01, ***p < 0.001.

To confirm this hypothesis, we inhibited proteasome activity with MG132 in PMN-MDSCs with Malata1 knockdown and then evaluated the p-STAT3 protein. The results indicated that Malat1 knockdown enhance the protein level of p-STAT3 after Malat1 knockdown, but this phenomenon was significantly weakened by inhibition of proteasome activity with MG132 (Fig. 5A). Further, The effect of Malat1 overexpression on the reduction of p-STAT3 was also significantly impaired after inhibiting the preoteasome (Fig. 5B). In addition, ubiquitination assay revealed that p-STAT3 protein ubiquitination significantly reduced in PMN-MDSCs exhibiting low expression of Malat1, whereas Malat1 overexpression could facilitate p-STAT3 ubiquitination (Fig. 5C, D). Together, these results demonstrate that Malat1 could inhibit STAT3 phosphorylation by enhancing p-STAT3 protein ubiquitination.

### The inhibition of STAT3 phosphorylation alleviates the immunosuppressive status in late sepsis

**Figure 6:**
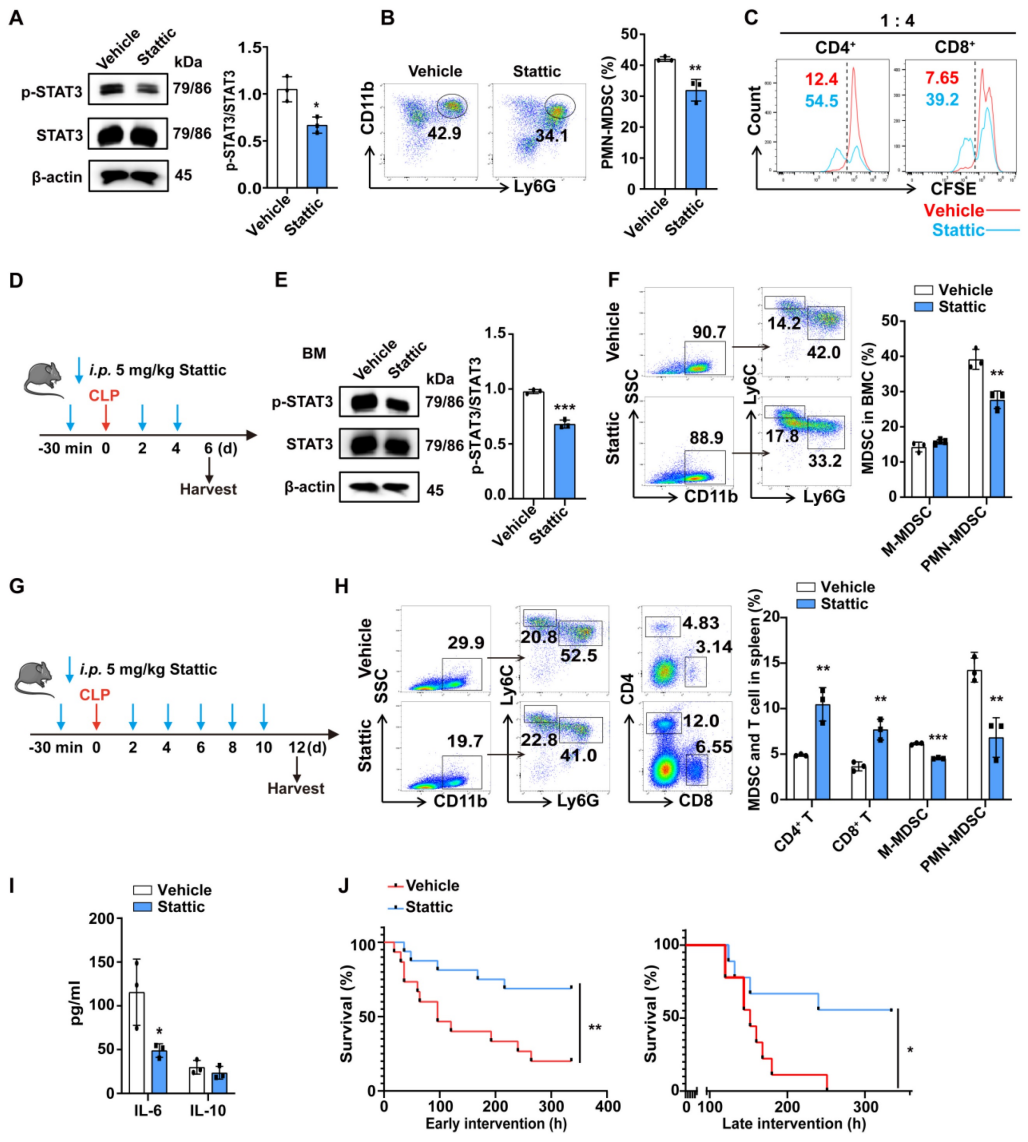
** Stattic improves survival in late sepsis by inhibiting the expansion and function of PMN-MDSCs.** (A, B) After 48 h of MDSC induction *in vitro*, Stattic or DMSO was added to the medium for another 24 h. (A) Representative flow cytometry plots (Left) and the statistical graph (Right) show the percentage of PMN-MDSCs in BMCs after inhibiting the STAT3 pathway. (B) p-STAT3 protein levels in BMCs (Left) after inhibiting the STAT3 pathway and quantification analysis (Right). (C) Induced PMN-MDSCs were treated with or without Stattic for 6 h, and then were co-cultured with CFSE-labeled splenocytes (1:4 ratio) for 3 days. T cell proliferation was detected by flow cytometry. (D) Schematic illustration of administration protocol in septic mice. (E) STAT3 protein phosphorylation assessment (Left) in BMCs of CLP day 6 mice and quantification analysis (Right). n=3. (F) Representative flow cytometry plots (Left) and the statistical graph (Right) show the percentage of PMN-MDSCs and M-MDSCs in BMCs of CLP mice after inhibiting the STAT3 pathway. n=3. (G) Schematic illustration of administration protocol in septic mice. (H) Representative flow cytometry plots (Left) and the statistical graph (Right) show the percentage of MDSCs and T cells in the spleen of CLP mice after inhibiting the STAT3 pathway. n=3. (I) CLP mice were injected with or without Stattic on day 12, and concentrations of IL-6 and IL-10 in plasma were detected by Elisa. n=3. (J) Fourteen-day survival curves of CLP mice treated with Stattic (n = 16) or DMSO (n = 15) starting before CLP (Left). 14-day survival curves of CLP mice treated with Stattic or DMSO. Starting on day 4 after CLP (Right). n=9. Data are presented as means ± SEM. *p < 0.05, **p < 0.01, ***p < 0.001.

Previous studies demonstrated that the inhibition of STAT3 tyrosine phosphorylation by Stattic prevented systemic inflammation and improved survival in experimental sepsis 40. Guha et al. reported that STAT3 inhibition induces Bax-dependent apoptosis in liver tumor MDSCs 41. To explore whether STAT3 inhibition can improve the immune status in late sepsis by affecting PMN-MDSCs, we first evaluated whether the inhibition of STAT3 phosphorylation with Stattic could affect the differentiation and function of PMN-MDSCs *in vitro*. The inhibition of STAT3 phosphorylation with Stattic was first confirmed by western blot analysis (Fig. 6A). The differentiation of PMN-MDSCs in BMCs was inhibited in the Stattic-treated group (Fig. 6B). The inhibitory function of PMN-MDSCs on T cell proliferation was also compromised in the Stattic-treated group (Fig. 6C).

Mice were intraperitoneally injected with Stattic or Vehicle at a dose of 5 mg·kg^-1^ body weight before and after CLP operation, as shown in Fig. 6D. BMCs were obtained 5 days later and the percentages of MDSCs were detected. We found that Stattic inhibited the phosphorylation of STAT3 and reduced the proportion of PMN-MDSCs but had no significant effect on M-MDSCs *in vivo* (Fig. 6E, F). In spleen, when treatment was extended to CLP day 10, Stattic not only reduced the proportion of both PMN-MDSCs and M-MDSCs but also increased the proportion of CD4^+^ T cells and CD8^+^ T cells (Fig. 6G, H). And plasma levels of IL-6 were reduced in late septic mice treated with Stattic, which may suggest an improvement in a persistent low-level inflammation (Fig. 6I). When we treated mice with Stattic, either early or delayed administration (starting on the fourth day after CLP) improved the survival of septic mice (Fig. 6J). Collectively, these results provide new evidence for therapeutic approaches targeting STAT3 to improve immune status in late sepsis.

## Discussion

In the present study, we found that Malat1 was continuously low expressed in BMCs during late sepsis, and Malat1 knockdown promoted the expansion and immunosuppressive function of PMN-MDSCs. In addition, Malat1 modulated PMN-MDSCs by inhibiting the stability of the p-STAT3 protein through the ubiquitin-proteasome system. Considering the protective effects of MDSCs in acute sepsis, we found that the adoptive transfusion of PMN-MDSCs in early sepsis not only promoted bacterial clearance but also inhibited the expansion of PMN-MDSCs in late sepsis. Additionally, the inhibition of STAT3 activation also improved the immunosuppressive state and survival in late sepsis by negatively affecting PMN-MDSCs.

PMN-MDSCs appear to be the dominant subset of MDSCs in septic children, as they account for more than 50% of patients' PBMCs in our data. Although there were considerable numbers of Gr1^+^CD11b^+^ cells in the BMCs of mice under physiological conditions, these CD11b^+^Ly6G^+^Ly6C^lo^ cells had no immunosuppressive function on T-cell proliferation. Their levels were reduced in the BMCs but increased in the circulating WBCs of CLP day 1 mice, when the levels of inflammatory cytokines were elevated. Therefore, we tend to consider these CD11b^+^Ly6G^+^Ly6C^lo^ cells as inactive PMN-MDSCs or other immune cells, such as neutrophils.

Currently, there is no appropriate definition of late sepsis. Based on organ damage, cytokine levels, and MDSCs, we suggested that CLP day 6 and later may be in the stage of immunosuppression. PMN-MDSCs from CLP day 6 mice did show significant T-cell proliferation inhibition. In mice subjected to CLP, MDSCs gradually infiltrate secondary lymphoid tissues, in which they represent as much as 10%-20% of all leukocytes 9. In spleen, MDSCs expand within 3-5 days, culminating after 10-14 days and staying high for at least 12 weeks 42. Our data are consistent with these phenomena. We provided a non-exhaustive overview of organ damage, inflammatory cytokine release, and MDSC changes during sepsis progression. Further research may be needed to elucidate the relationship between MDSCs and PICS, and how MDSC expansion is related to sepsis-induced organ dysfunction.

Although the early host response is recognized immediately at the site of infection by resident innate cell populations, the recruitment of additional innate immune cells from the BM is almost immediate 43,44. The product of a sepsis-induced cytokine milieu is a preferential differentiation toward myelopoiesis, and the BM is composed of nearly 95% myeloid cells within several days of sepsis 9. We found that the gene expression profile of BMCs changed dramatically with sepsis progression. Increasing evidence suggests that lncRNAs play critical roles in a diverse set of biological processes, including embryogenesis, proliferation, neurogenesis, differentiation, and stem cell pluripotency. Further studies have shown that lncRNAs participate in the regulation of immune reactions and the pathoetiology of several immune-related disorders, including sepsis 26,45. Malat1 is a lncRNA whose participation in sepsis pathophysiology has been well investigated. Although some studies have shown elevated Malat1 expression in septic patients and animal/cell models 26,46, few have concluded that Malat1 is decreased in sepsis 47. Here, we demonstrated a variable trend in Malat1 expression in BMCs and circulating WBCs of septic mice. Zhou et al. found that total MDSCs increase following Malat1 knockdown in PBMCs from patients with lung cancer 28. Our data showed that persistent low Malat1 expression in late sepsis promotes the expansion and activation of PMN-MDSCs by maintaining the phosphorylation of STAT3. STAT3 is associated with the expansion and immunosuppressive function of PMN-MDSCs 15. In mice exposed to CLP, STAT3 in BMCs was activated at a fairly early stage, due to the influence of various factors such as inflammatory cytokines. However, the activation of STAT3 remained significant after the regression of inflammatory cytokines, which may be regulated by Malat1-mediated ubiquitination. Persistent MDSC expansion in sepsis can be detrimental, as it both propagates persistent inflammation and dampens the adaptive immune response through T cell suppression. The development of MDSCs induced by sepsis still has unknown aspects. MDSCs account for a high proportions of BMCs in late septic mice, and our regular gene expression profile of BMCs may also reflect the development process of MDSCs. Future transcriptomic analyses of MDSCs in different sepsis stages can be conducted to search for lineage genes, which may provide therapeutic targets.

Until now, the separation of PMN-MDSCs from other polymorphonuclear neutrophils (PMNs) in the same mouse was not possible, due to an absence of specific markers. In blood samples from cancer patients, PMN-MDSCs can be separated from other PMNs by gradient centrifugation. This has allowed the identification of a distinct transcriptomic profile of PMN-MDSCs and the identification of LOX-1 as a marker of human PMN-MDSCs 13. Recent studies have established a gene signature of PMNs and PMN-MDSCs at the single-cell transcriptomes level and identified CD84 as a surface marker for the improved detection and enrichment of MDSCs in breast cancers 48. We found that Malat1 knockdown increased the percentage of CD84^+^PMN-MDSC. However, we did not detect intramedullary CD84^+^ MDSC production in late septic mice (Fig. S5D-F). Therefore, CD84 may be not suitable for use as an indicator of MDSC generation and capacity in septic mice.

The expression of CD244 has been confirmed to be useful for identifying PMN-MDSCs 30. We did find the increased expression of CD244 in PMN-MDSCs of late septic mice, indicating that it may be more suitable for sepsis-related studies. Another study characterized the heterogeneity of PMNs in cancer by using single-cell RNA-seq, single-cell mass cytometry by time-of-flight (CyTOF), flow cytometry, and functional analysis and suggested that PMN-MDSCs in mice could be identified based on CD14 expression 49. However, no detectable CD14 expression was observed in PMNs from healthy donors or cancer patients 49. Hence, there is still no recognized unique surface marker for MDSC identification. In addition, MDSCs from sepsis survivors potentially differ from phenotypically similar cells reported in cancer and autoimmunity 50. For example, Arg-1 and PD-L1 have been suggested to have an important role in MDSC function in cancer, but their expression is not significantly increased in the MDSC subsets of late septic patients 51. At present, the function of MDSCs in different studies has been determined by its inibition on T cells of splenocytes 52-54. It may be necessary to re-examine whether sepsis-induced MDSCs have unique features either regarding development or function.

Several strategies have been proposed to treat sepsis by targeting MDSCs. For example, LXR agonists improve the survival of septic mice by boosting the apoptosis of splenic MDSCs 15,55. The adoptive transfer of day 10 MDSCs to septic mice has been proven to attenuate peritoneal cytokine production, increase bacterial clearance, and dramatically improve survival rates 20. Zhou et al.^56^ have found that MDSCs differentiated from Ly6G^-^ BMCs suppressed T-cell proliferation more strongly, whereas cells differentiated from Ly6G^+^ BM were non-immunosuppressive. We hypothesized that when PMN-MDSCs treatment altered the composition of BMCs in early sepsis (more Ly6G^+^ cells retained), the proportions and function of PMN-MDSC production in late sepsis might be different. Our data demonstrated that the adoptive transfusion of PMN-MDSCs in early sepsis inhibited the subsequent expansion of MDSCs in the host BM and circulation. In 2010, when Geber et al. 40 treated experimental septic mice, they found that Stattic prevented systemic inflammatory responses and improved survival rates. Stattic binds specifically to the STAT3 SH2 domain and inhibits its dimerization and DNA-binding, thereby inhibiting STAT3 activation and nuclear translocation. Stattic selectively inhibits the phosphorylation of STAT3 at Tyr705 but has little inhibitory effect on its phosphorylation at Tyr701 or the phosphorylation of JAK1, JAK2, and other molecules. Stattic was able to induce apoptosis in liver tumor-derived MDSC in a Bax-dependent manner 41. Considering the Malat1-STAT3-PMN-MDSC axis, we speculated that inhibiting STAT3 activation may have an impressive therapeutic effect on late sepsis, which was supported by our results. We have not investigated the effect that by altering Malat1 expression *in vivo* may have on sepsis. Chen et al. 46 found that Malat1 null mice have higher survival rates when insulted by CLP because of the activation of the antioxidant pathway. These results imply the complex role of Malat1 at different stages of sepsis.

There are a few limitations to this research. First, we did not isolate the progenitor cells of PMN-MDSC, hence, we cannot determine at what stage of cell development Malat1 affects PMN-MDSC expansion. Phosphorylation and ubiquitination are pervasive post-translational modifications that impact all processes inside eukaryotic cells. Our understanding of the molecular features that allow phosphorylation to control protein ubiquitination and ubiquitin to control phosphorylation has recently begun to build 57. However, in this paper, we only examined the effect of ubiquitination on p-STAT3 degradation and did not consider whether there was any interaction between ubiquitin and phosphorylation. In patient studies, it is difficult to define early and late sepsis because some patients have been treated elsewhere before arriving at the ICU.

In conclusion, our data provide novel mechanistic information regarding Malat1 regulation of PMN-MDSCs in sepsis. To our knowledge, this is the first study to link the reduction of Malat1 and ubiquitination degradation of p-STAT3 causing the expansion and activation of PMN-MDSCs. This preclinical study not only offers a mechanistic basis for PMN-MDSC accumulation during sepsis progression but also defines potential strategies for limiting PMN-MDSCs in the management of sepsis.

## Supplementary Material

Supplementary figures and tables.

## Abbreviations

Arg-1: Arginase 1
BMC: Bone marrow cell
CCL2: C-C motif chemokine ligand 2
CLP: Cecal ligation and puncture
GM-CSF: Granulocyte macrophage-colony stimulating factor
TNF-α: Tumor necrosis factor alpha
iNOS: Inducible nitric oxide synthase
LncRNA: Long non-coding RNA
MALAT1: Metastasis associated lung adenocarcinoma transcript 1
MDSC: Myeloid-derived suppressor cell
M-MDSC: Monocytic-MDSC
PMN-MDSC: Polymorphonuclear-MDSC
PICS: Persistent inflammation, immunosuppression, and catabolism syndrome
ROS: Reactive oxygen species
STAT3: Signal transducers and activators of transcription 3
SOCS3: Suppressors of cytokine signaling 3
