# Supplementary material for: Malat1 regulates PMN-MDSC expansion and immunosuppression through p-STAT3 ubiquitination in sepsis
Source: Int J Biol Sci. 2024 Feb 11;20(4):1529–46. doi: 10.7150/ijbs.92267 (PMC10878150; doi:10.7150/ijbs.92267)
Supplement: Supplementary file 1 — Supplementary figures and tables. [file ijbsv20p1529s1.pdf]

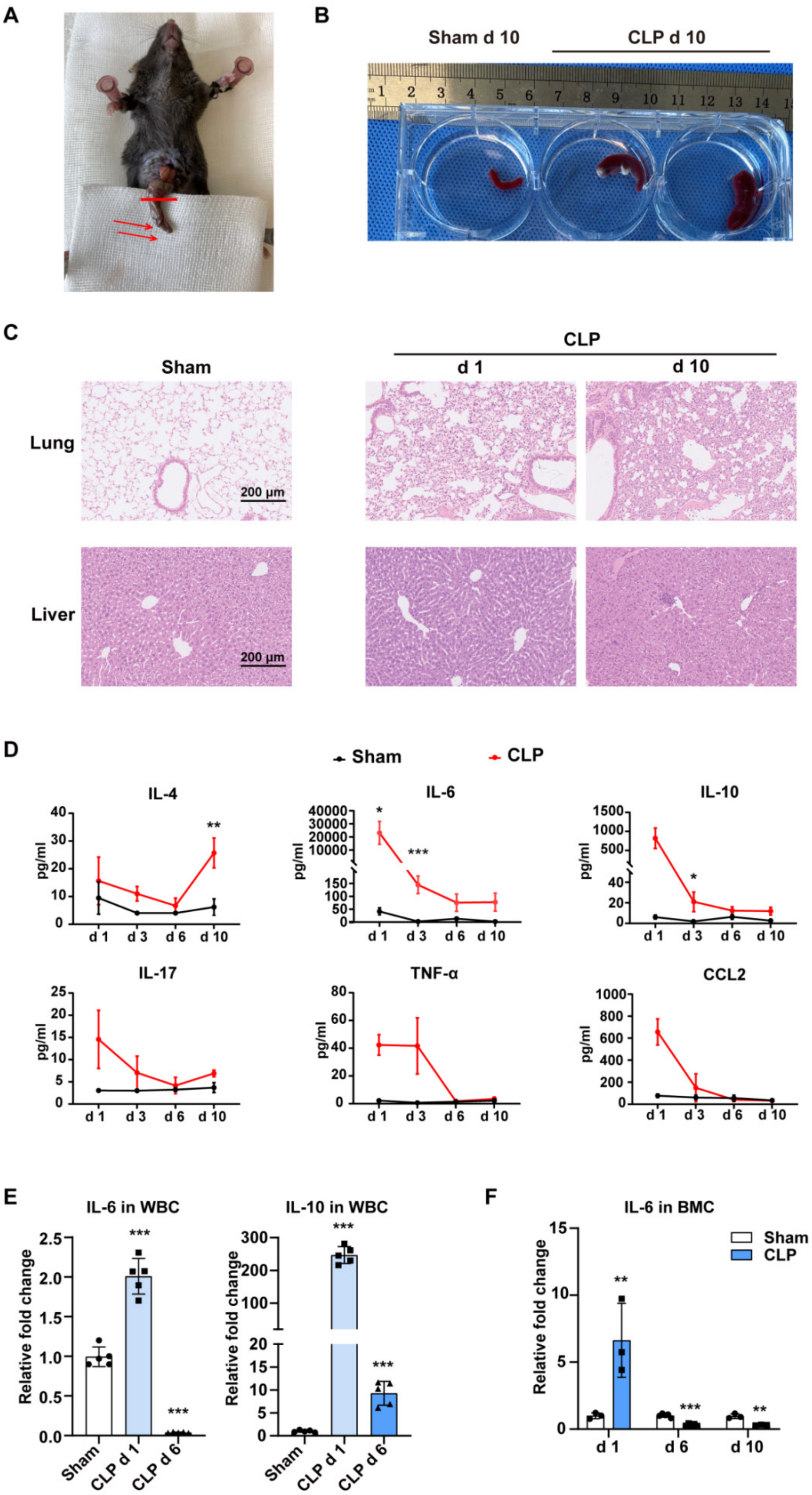

Fig. S1 CLP-induced sepsis imitated altered immune status

(A) Sites of cecum ligation and puncture in mice. (B) Spleen size of sham and CLP

mice on day 10. (C) Representative HE staining images of lung and liver lesions of sham, CLP d 1, and CLP d 10 mice. Scale bar, 200 μm. (D) Plasma cytokines and chemokines were measured by the Luminex assay at different times after CLP surgery. For each time point of the sham group, n=3; for the CLP group, n=5–8. (E) RT-qPCR analysis of IL-6 (Left) and IL-10 (Right) expression in WBCs of sham and CLP mice. n=5. (D) RT-qPCR analysis of IL-6 expression in BMCs of sham and CLP mice. n=3-5. Data are presented as means ± SEM. *$p < 0.05$, **$p < 0.01$, ***$p < 0.001$.

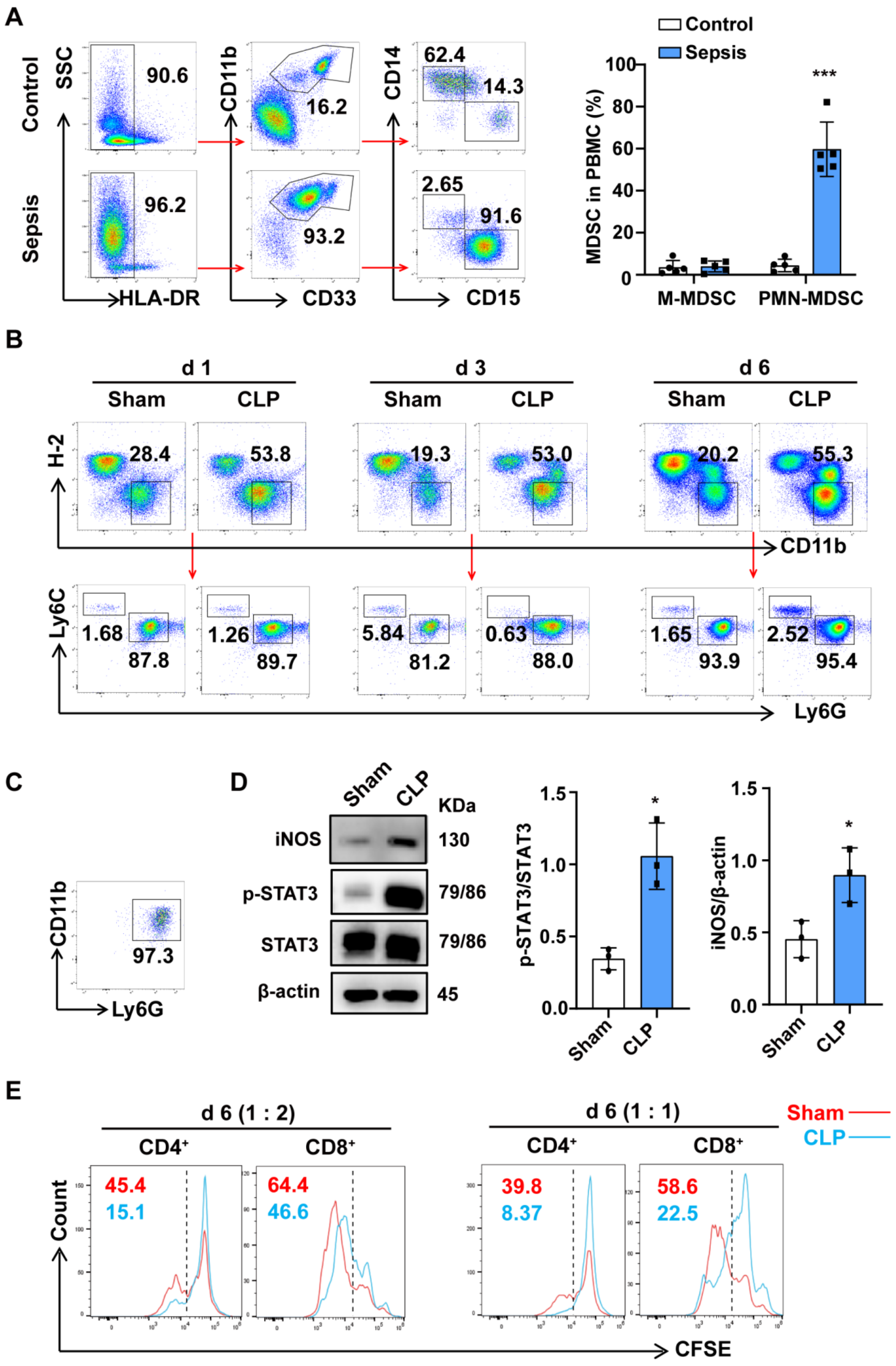

Fig. S2 PMN-MDSCs expand and persist in the late stage of sepsis

(A) Representative flow cytometry plots (Left) and the statistical graph (Right) show

the percentage of HLA-DR$^-$CD11b$^+$CD33$^+$CD14$^-$CD15$^+$ PMN-MDSCs and HLA-DR$^-$CD11b$^+$CD33$^+$CD14$^+$CD15$^-$ M-MDSCs in PBMCs of control and patients with sepsis or septic shock. n=5. (B) Representative dot plots show the percentage of MDSCs in WBCs of sham and CLP mice. (C) Representative dot plot shows the sorting efficiency of CD11b$^+$Ly6G$^+$cell. (D) p-STAT3 and iNOS protein expression in PMN-MDSCs isolating from BMCs of sham and CLP mice on day 6 post-surgery (Left) and quantification analysis (Right). n=3. (E) CD11b$^+$Ly6G$^+$ cells from BMCs of sham and CLP mice on day 6 post-surgery were co-cultured with CFSE labeled splenocytes in a ratio of 1:2 or 1:1 for 3 days, and anti-CD3 plus anti-CD28 antibodies were added to the culture to activate T cells. T cells' proliferations were detected by flow cytometry. Representative flow cytometry plots of CFSE positive and low-positive T cells gated on CD4 and CD8 are shown. Data are presented as means ± SEM. *$p < 0.05$, ***$p < 0.001$.

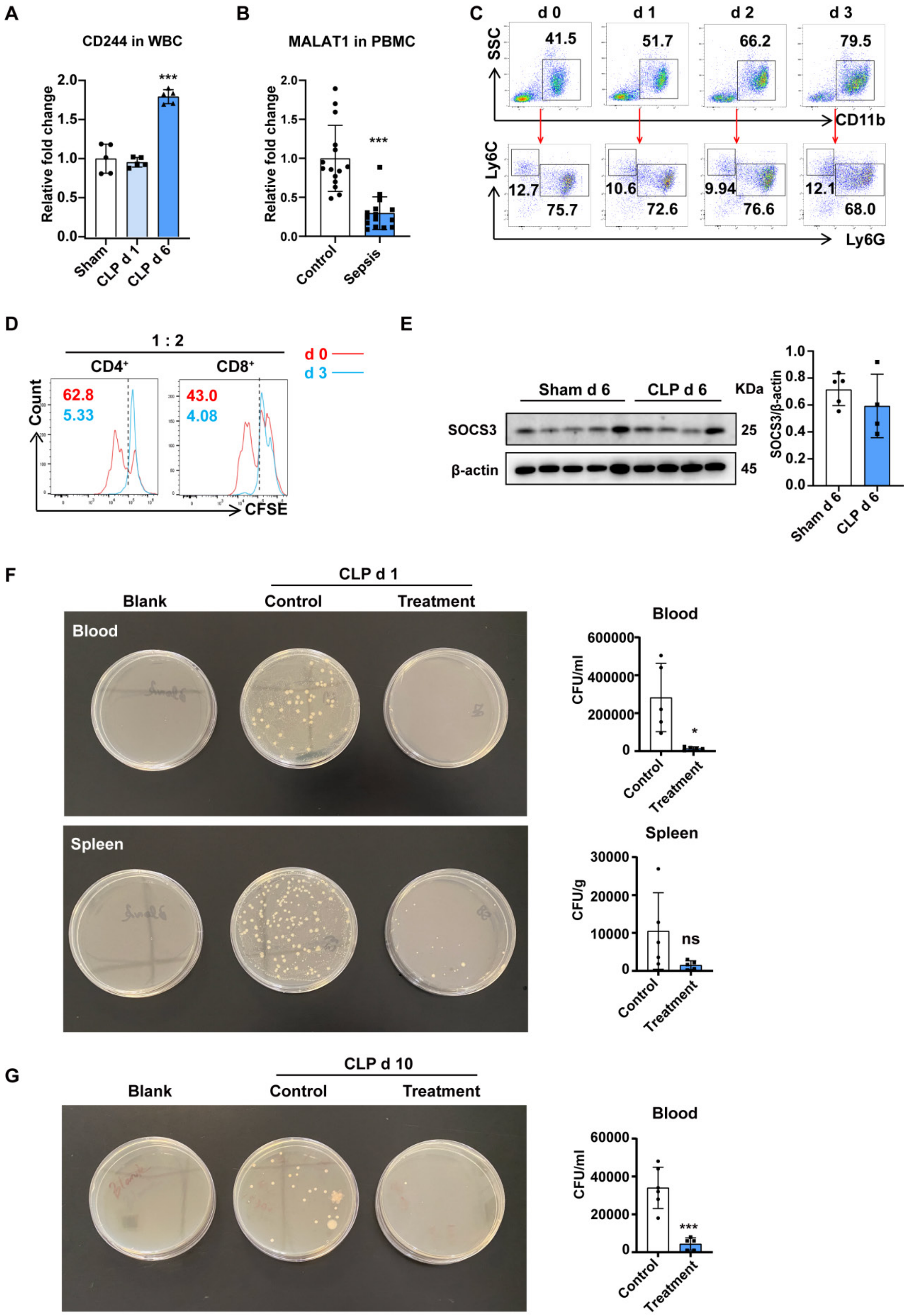

Fig. S3 Expansion and function of GM-CSF and IL-6-induced PMN-MDSCs in vitro

(A) RT-qPCR analysis of CD244 expression in WBCs of sham and CLP mice. n=5. (B) RT-qPCR analysis of MALAT1 expression in PBMCs of control and patients with sepsis or septic shock. n=15. (C–D) Fresh BMCs were isolated and cultured with GM-CSF plus IL-6 in vitro. (C) Representative flow cytometry plots show the percentage of MDSCs' subsets in BMCs after inducing. (D) $CD11b^{+}Ly6G^{+}$ cells from fresh or induced BMCs on day 3 were co-cultured with CFSE labeled splenocytes in a ratio of 1:2 for 3 days. T cells' proliferations were detected by flow cytometry. (E) SOCS3 protein expression in BMCs of sham and CLP mice on day 6 post-surgery (Left) and quantification analysis (Right). n=4-5. (F–G)Among treatment group, mice were received 1×10^{6} PMN-MDSCs *i.v.* immediately after CLP. Mice in control group were treated with equal volumes of PBS. (F) Results of bacterial counts in whole blood and spleen 1 day after CLP and adoptive transfer are expressed as CFU per mL of blood or CFU per gram of spleen. n=4-5. (G) Results of bacterial counts in whole blood 10 days after CLP and adoptive transfer are expressed as CFU per ml of blood. n=5. Data are presented as means ± SEM. *$p < 0.05$. ***$p < 0.001$.

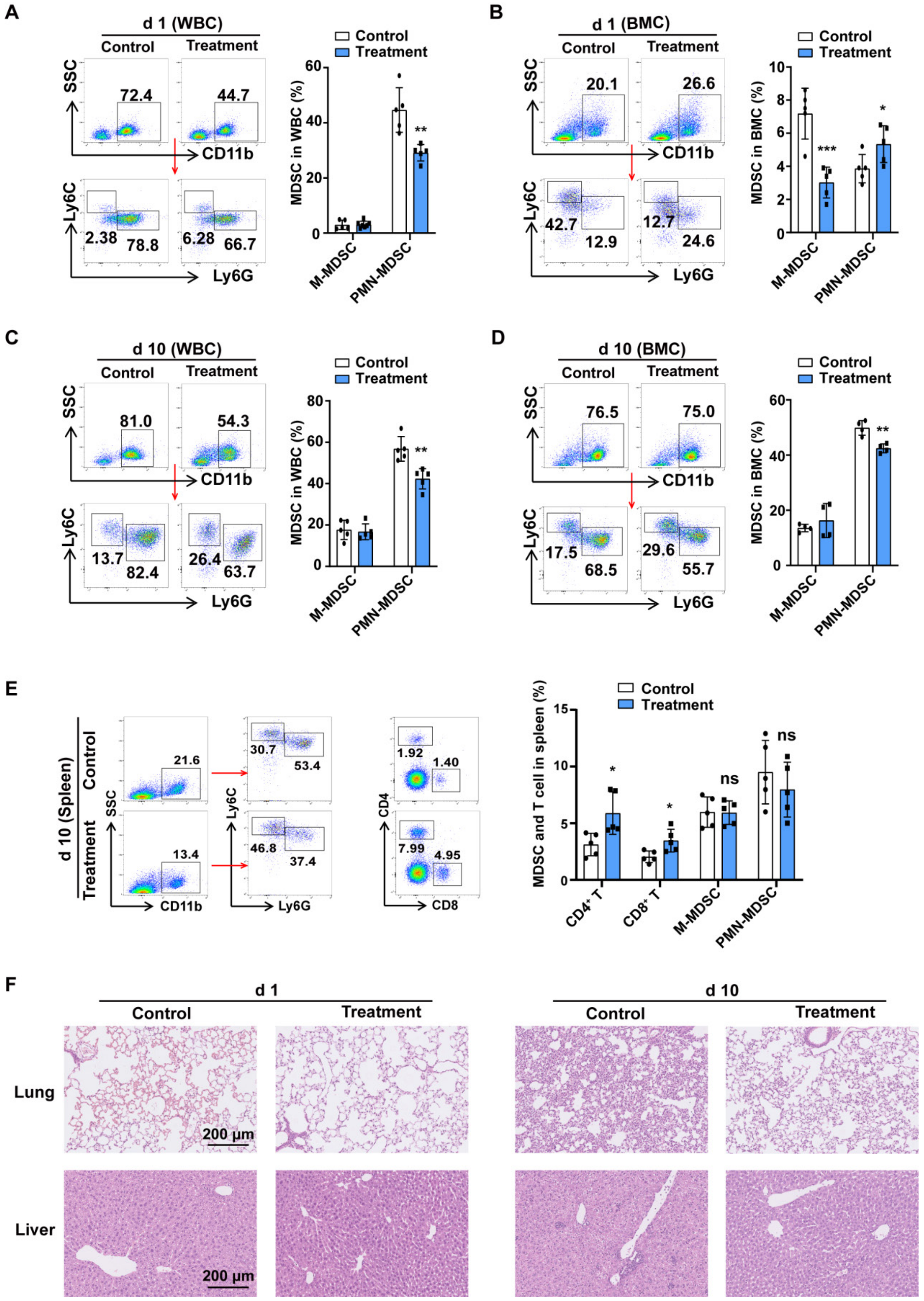

Fig. S4 Adoptively transferred PMN-MDSCs in early sepsis confer protection

Among the treatment group, mice received $1{\times}10^6$ PMN-MDSCs *i.v.* immediately after CLP. Mice in the control group were treated with equal volumes of PBS. (A–B) One

day after CLP and adoptive transfer, the therapeutic effects of PMN-MDSCs were examined. n=5. (A) Representative flow cytometry plots (Left) and the statistical graph (Right) show the percentage of MDSC subsets in WBCs. (B) Representative flow cytometry plots (Left) and the statistical graph (Right) show the percentage of MDSC subsets in BMCs. (C–E) On day 10 after CLP and adoptive transfer, the therapeutic effects of PMN-MDSCs were examined. n=5. (C) Representative flow cytometry plots (Left) and the statistical graph (Right) show the percentages of MDSC subsets in WBCs. (D) Representative flow cytometry plots and the statistical graph show the percentage of MDSC subsets in BMCs. (E) Representative flow cytometry plots and the statistical graph show the percentage of MDSC subsets and T cells in spleens. (F) Representative HE staining images of lung and liver lesions of each group. Scale bar, 200 μm. Data are resented as means ± SEM. *$p < 0.05$, **$p < 0.01$, ***$p < 0.001$.

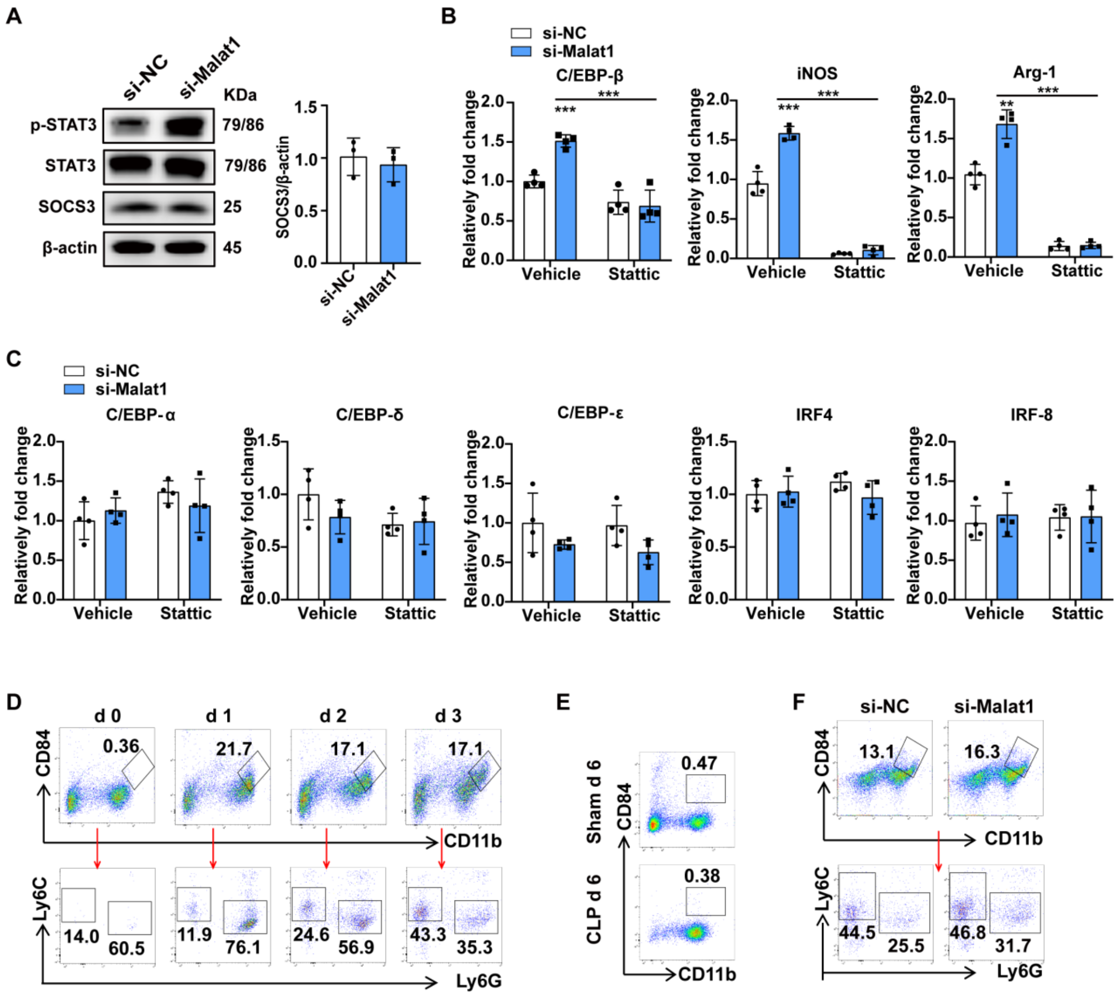

Fig. S5 Malat1 inhibits the expansion and immunosuppressive function of PMN-MDSC by reducing STAT3 phosphorylation

(A) SOCS3 protein expression in BMCs after Malat1 knockdown (Left) and quantification analysis (Right). n=3. (B-C) RT-qPCR analysis of C/EBPα, C/EBP β , C/EBP δ , C/EBPε, IRF4, IRF8, ARG-1 and iNOS mRNA levels in group of si-NC, si-Malat1, si-NC+stattic and si-Malat1+stattic. (D) Representative flow cytometry plots show the percentage of CD84$^+$MDSCs in BMCs after GM-CSF plus IL-6 inducing. (E) Representative flow cytometry plots show the percentage of CD84$^+$ MDSC in BMCs of sham and CLP mice on day 6 post-surgery. (F) Fresh BMCs were transfected with Malat1-specific or scramble siRNA and ASO for 36 h, and then

treated with GM-CSF (20 ng/ml) and IL-6 (20 ng/ml) for another 36 h. Representative dot plots show the percentage of $CD84^{+}$MDSCs' subsets after Malat1 knockdown. Data are presented as means ± SEM. ***p* < 0.01, ****p* < 0.001.

Supplementary tables

Supplementary Table 1 Demographic data of patients with and without Sepsis

Parameter	Control (n=15)	Sepsis (n=15)	*p* value
Age[a]			1
< 1	5	6	
1–5	10	9	
Gender[a]			1
Male	10	11	
Femal	5	4	
Weight[b]			0.88
	12.5 (8.25, 12.5)	10 (9.6, 14)	
Diagnosis			
Sepsis/Septic shock	0	15	
Inguinal hernia	15	0	
Discharge service			
Recovery	15	4	
Death/Automatically discharge	0	11	

a. Data are given as number of patients in group. Group comparisons were made using Fisher exact test.

b. For variables with skewed distribution, data are given as median values and inter quartile range. And the Wilcoxon rank sum test was used.

Supplementary Table 2 Sequence of primers and Malate1 smart silencer

Name	Usage	Sequence (5’-3’)
Mus Actb	F	GTGACGTTGACATCCGTAAAGA
	R	GCCGGACTCATCGTACTCC
Mus Arg-1	F	TGTCCCTAATGACAGCTCCTT
	R	GCATCCACCCAAATGACACAT
Mus Cd244	F	CCAGCCGATTTTGTCTTCTT
	R	TAGTCCTTCCTACCCCAATTTCC
Mus Cebpa	F	CAAGAACAGCAACGAGTACCG
	R	GTCACTGGTCAACTCCAGCAC
Mus Cebpb	F	CAACCTGGAGACGCAGCACAAG
	R	GCTTGAACAAGTTCCGCAGGGT
Mus Cebpd	F	CGACTTCAGCGCCTACATTGA
	R	GAAGAGGTCGGCGAAGAGTT
Mus Cebpe	F	GCAGCCACTTGAGTTCTCAGG
	R	GATGTAGGCGGAGAGGTCGAT
Mus Il6	F	TAGTCCTTCCTACCCCAATTTCC
	R	TTGGTCCTTAGCCACTCCTTC
Mus Il10	F	AGCCTTATCGGAAATGATCCAGT
	R	GGCCTTGTAGACACCTTGGT
Irf4	F	CTTTGAGGAATTGGTCGAGAGG
	R	GAGAGCCATAAGGTGCTGTCA

Irf8	F	GGCTGCATGAGCGAAGTTC
	R	CTCCTCTTGGTCATACCCATGTA
Mus Malat1	F	GGGAGTGGTCTTAACAGGGAGGAG
	R	GTGCCAACAGCATAGCAGTACACG
Mus Nos2	F	GTTCTCAGCCCAACAATACAAGA
	R	GTGGACGGGTCGATGTCAC
Homo GAPDH	F	CCATCAATGACCCCTTCATTGACC
	R	GAAGGCCATGCCAGTGAGCTTCC
Homo MALAT1	F	GTGATGCGAGTTGTTCTCCG
	R	CTGGCTGCCTCAATGCCTAC
Malat1 overexpression	Primer1	GTTTAAACGGGCCCTCTAGACAGGCATTCAGGCAGCGAGAGCAGAGC
	Primer2	CAGCGGTTTAACTATCTAGATGAGAGATATTTAGTTTTTATTTCATAAAATC
Malat1 smart silencer	siRNA1	GTGAATGAGTGATAAGTAA
	siRNA2	GGTGCAGTGTGCCAATGTT
	siRNA3	GCTCAGGACTTTGCATATA
	ASO1	CGAACACTTAAGTGGACCAG
	ASO2	GCTAGCATCTTAGTGGAAGC
	ASO3	CCAAGGTCCTGTGGGTTGCA

Supplementary Table 3 Antibodies

**Antibodies**	**Source**	**Identifier**
β-Actin (13E5) Rabbit mAb	CST	Cat#4970S; RRID:AB_2223172
Arginase-1 Polyclonal antimody	Proteintech	Cat#16001-1-AP; RRID: AB_2289842
C/EBP β (LAP) Antibody	CST	Cat#3087S; RRID: AB_2078052
iNOS Polyclonal antimody	Proteintech	Cat#22226-1-AP; RRID: AB_2879038
SOCS3 Antibody	Affinity	Cat#DF6133; RRID: AB_2838100
Normal Rabbit IgG	CST	Cat#2729S; RRID: AB_1031062
Phospho-Stat3 (Tyr705) (D3A7) Rabbit mAb	CST	Cat#9145S; RRID: AB_2491009
Stat3 (79D7) Rabbit mAb	CST	Cat#4904S; RRID: AB_3083660
Ubiquitin (E4I2J) Rabbit mAb	CST	Cat#43124S; RRID: AB_2799235
